# Supplementary material for: Self-vectoring electromagnetic soft robots with high operational dimensionality
Source: Nat Commun. 2023 Jan 12;14:182. doi: 10.1038/s41467-023-35848-y (PMC9837125; doi:10.1038/s41467-023-35848-y)
Supplement: Supplementary file 1 — Supplementary Information [file 41467_2023_35848_MOESM1_ESM.pdf]

Supplementary Information Files for

Self-vectoring electromagnetic soft robots with high operational dimensionality

Wenbo Li[1,2]†*, Huyue Chen[3]†, Zhiran Yi[1], Fuyi Fang[1], Xinyu Guo[1], Zhiyuan Wu[1], Qiuhua Gao[1], Lei Shao[3]*, Jian Xu[2], Guang Meng[1], Wenming Zhang[1]*

*Corresponding author. E−mail: wenboli@tongji.edu.cn, lei.shao@sjtu.edu.cn, wenmingz@sjtu.edu.cn
†These authors contributed equally to this work

This PDF file includes:

1. Supplementary Figures 1 to 34
2. Supplementary Tables 1 to 4
3. Supplementary Notes
4. Supplementary References

Other Supplementary Materials for this manuscript include the following:

Supplementary Movies 1 to 19

1. Supplementary Figures

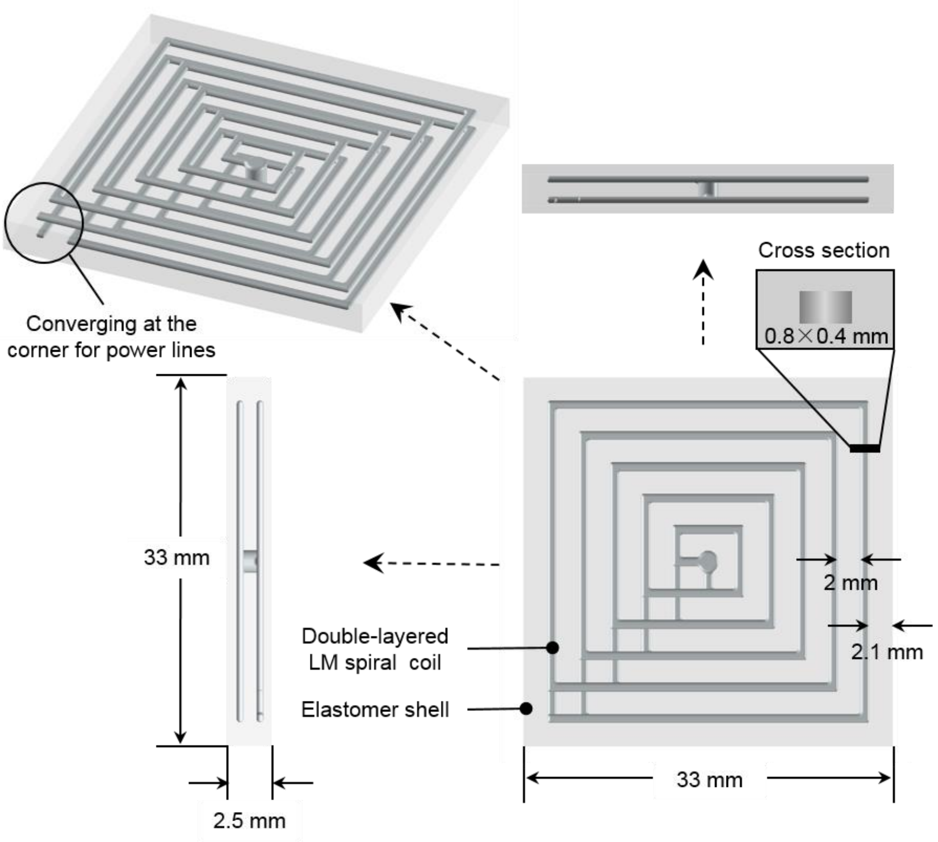

Supplementary Figure 1. The detailed structure and dimension of the soft vertical flux actuators (module V) for SESRs.

There are 5 loops of double-layered LM coils embedded in the elastomer shell. The two layered LM coils are connected at their centers and have the same direction of spiral. The two terminals of the holistic LM coil are designed to converge at the same corner of the actuators and connect to the power source through soft twisted-pair wires. By this method, the effect of the tethered control on the actuators when subject to external magnetic field can be minimized.

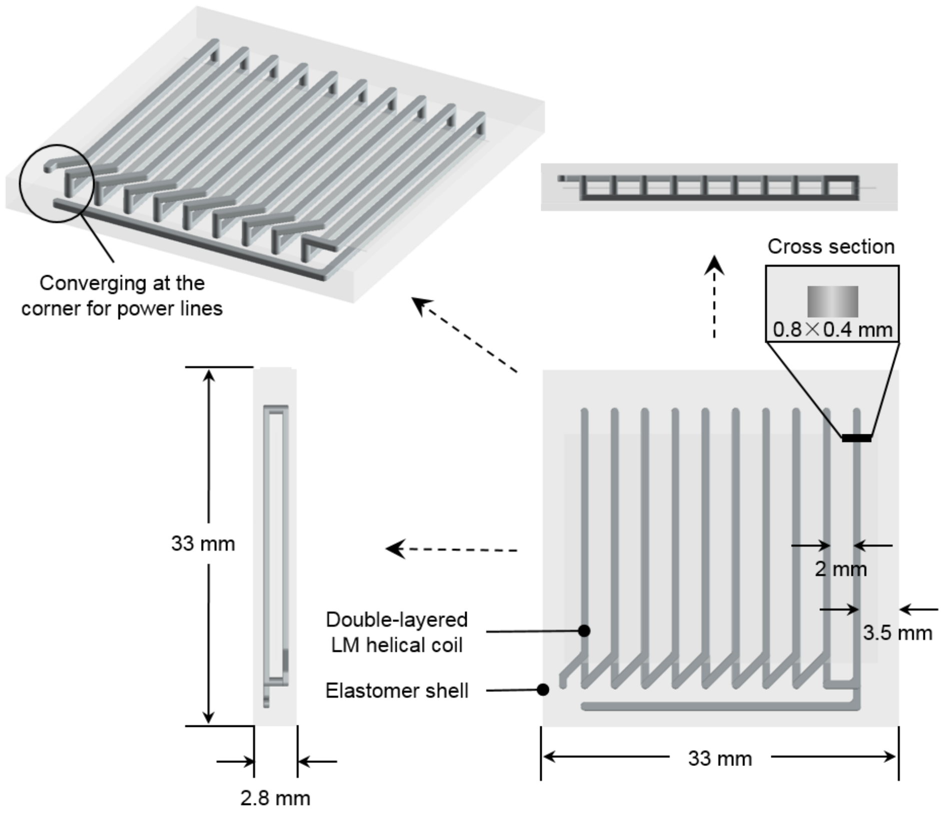

Supplementary Figure 2. The detailed structure and dimension of the soft inflatable horizontal flux actuators (module H) for SESRs.

The two terminals of the holistic LM coil are also designed to converge at the same corner of the actuators and connect to the power source through soft twisted-pair wires for module H.

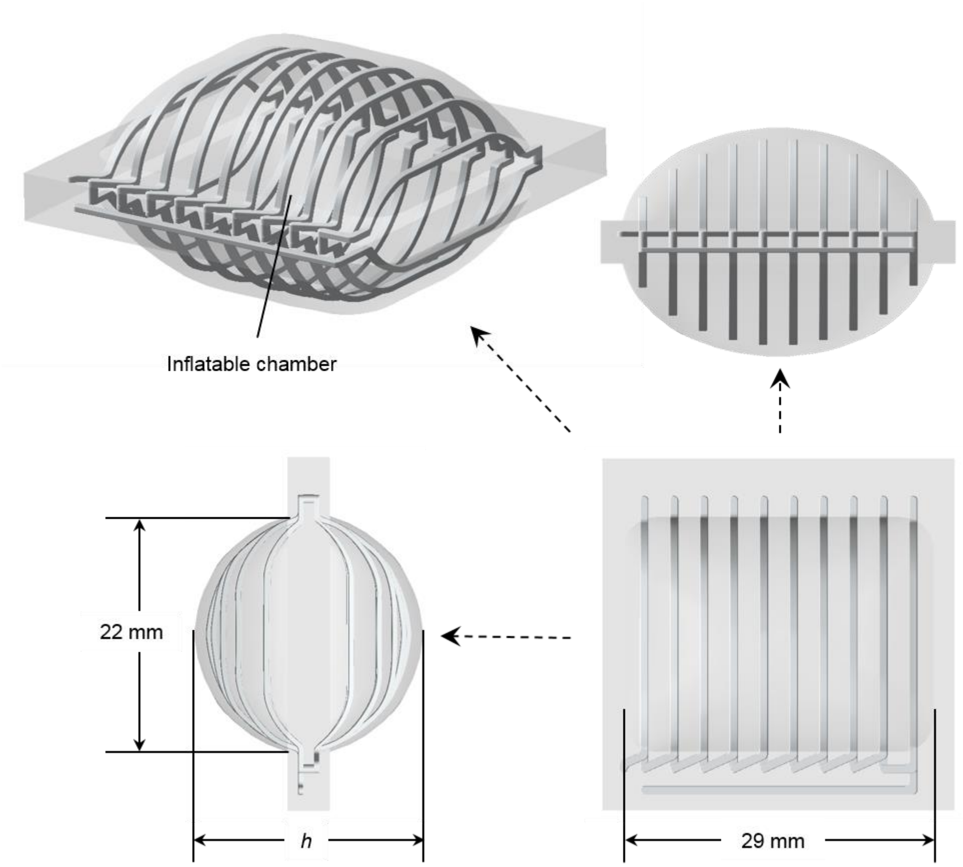

Supplementary Figure 3. The inflation configuration and dimension of the module H.

There are ten circles of LM coils embedded in the elastomer shell. They are connected in series in sequence and can be expanded by the interior inflatable chamber.

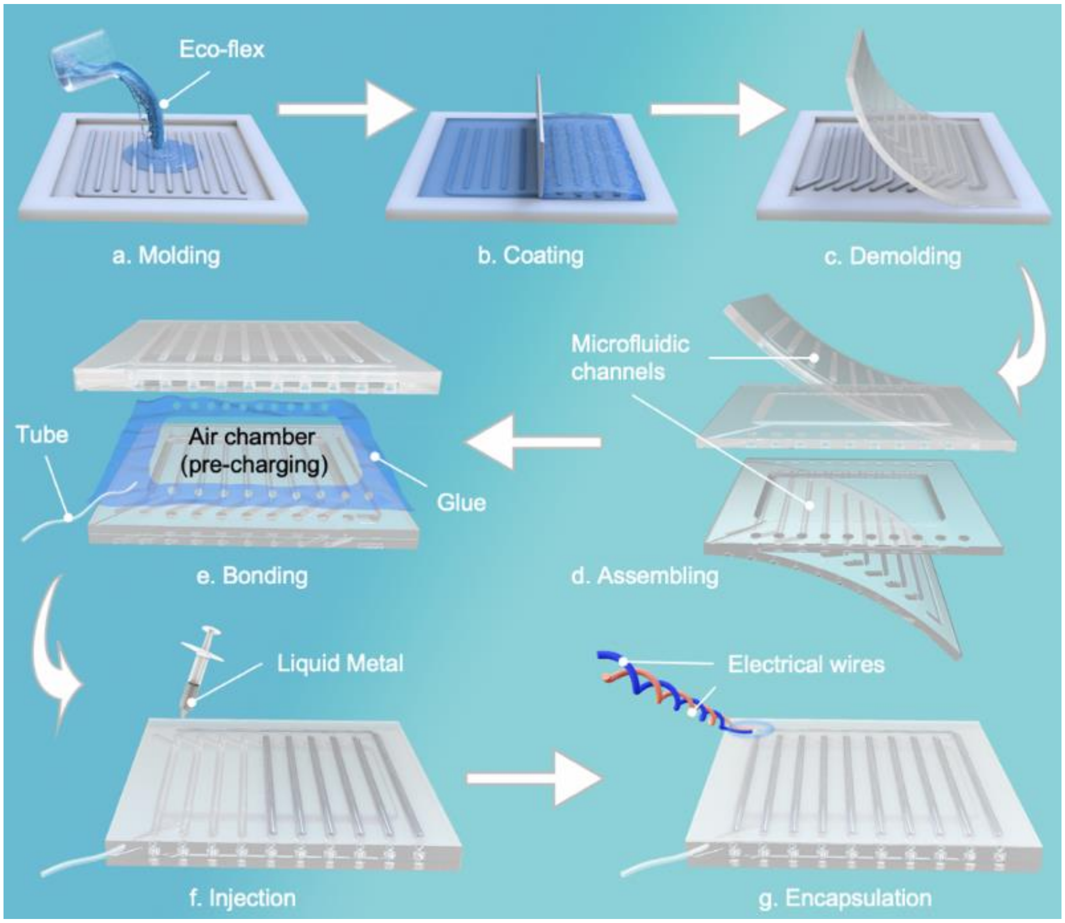

Supplementary Figure 4. Fabrication process of the module H.

a Molding and casting the super-soft shell with Eco-flex 30. The components with various micro-channels were fabricated with a high-precision Objet30 Connex3 PolyJet 3D printer. **b** Coating the liquid phase silicone rubber with a thin piece of glass. **c** Demolding the stretchable membranes after curing at room temperature. **d** Assembling all layers into two groups separately. All grooves and connecting holes are aligned to form semi-closed microchannels. **e** Bonding the upper and lower parts with the specific glue Sil-poxy. Before applying a layer of ultra-thin glue, we covered the selected part with a template to construct the middle chamber, connecting holes, and an air tube in advance. **f** Injecting the liquid metal (LM, EGaInSn) into the micro-channels. A small hole is opened at the other end of the channel to exhaust the air and prevent the rapid oxidation of LM. **g** Encapsulating the whole actuator. A set of soft twisted-pair electrical wires are used to eliminate the interference in the signal wire (The electrical wire will generate Lorentz force in the external magnetic field).

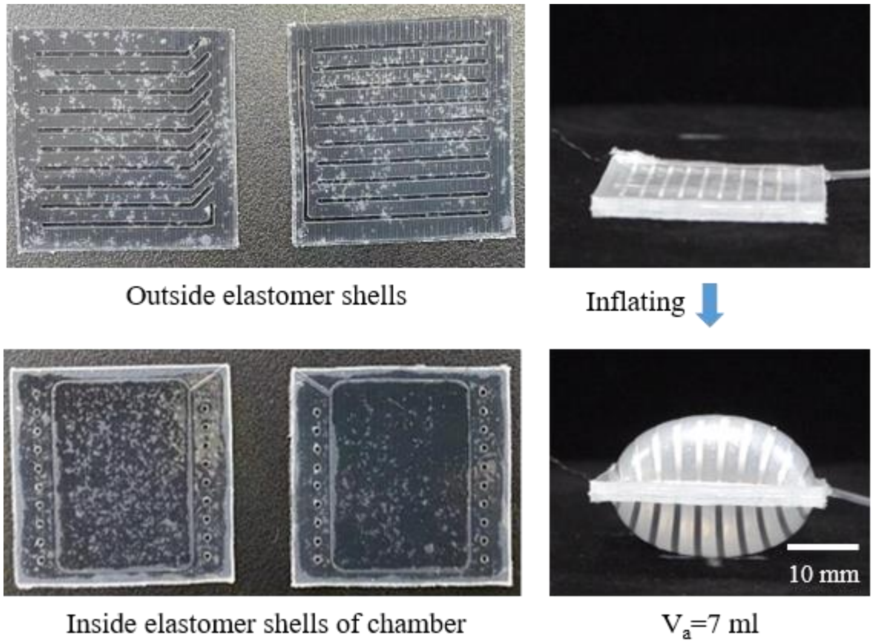

Supplementary Figure 5. Components of elastomer shells and the inflating deformation of module H.

There are two groups of soft shells for constructing module H. The outside elastomer shells contain the microchannels for flowing the LM, and the inside elastomer shells are the sealing layers for the outside shells and the chamber walls. The LM lines in the microchannels of the outside shells are connected through the abreast holes of the inside shells to form the interconnected LM coil. The module H is soft and its profile can be reconfigured by inflation, as well as the embedded LM helical coil.

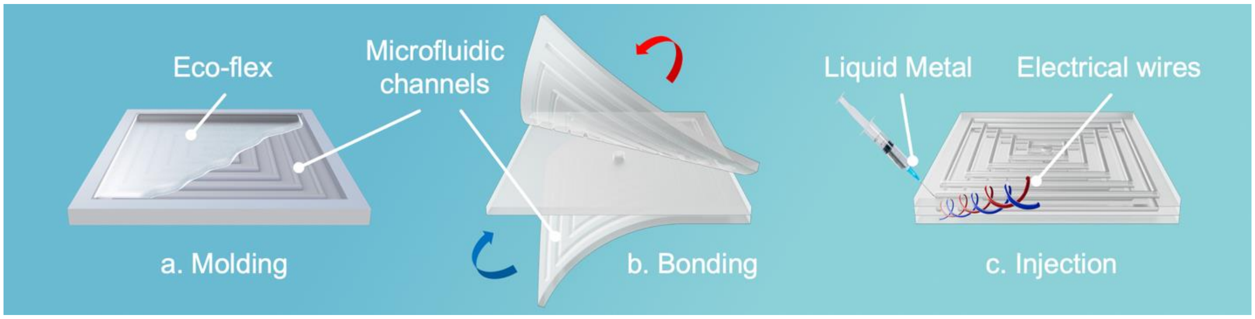

Supplementary Figure 6. Fabrication process of the module V.

a Molding, casting, and coating the super-soft shell with Eco-flex 30. **b** Demolding, assembling, and bonding three stretchable membranes to form microchannels inside. The microchannels of the upper and lower layers are consistent but rotated by 90 degrees; The middle layer only has a hole in the middle, which is used to connect all the microchannels. **c** Injecting the liquid metal, and encapsulating the whole actuator. Like module H, twisted-pair electrical wires are used to counteract each other's Lorentz force under a magnetic field.

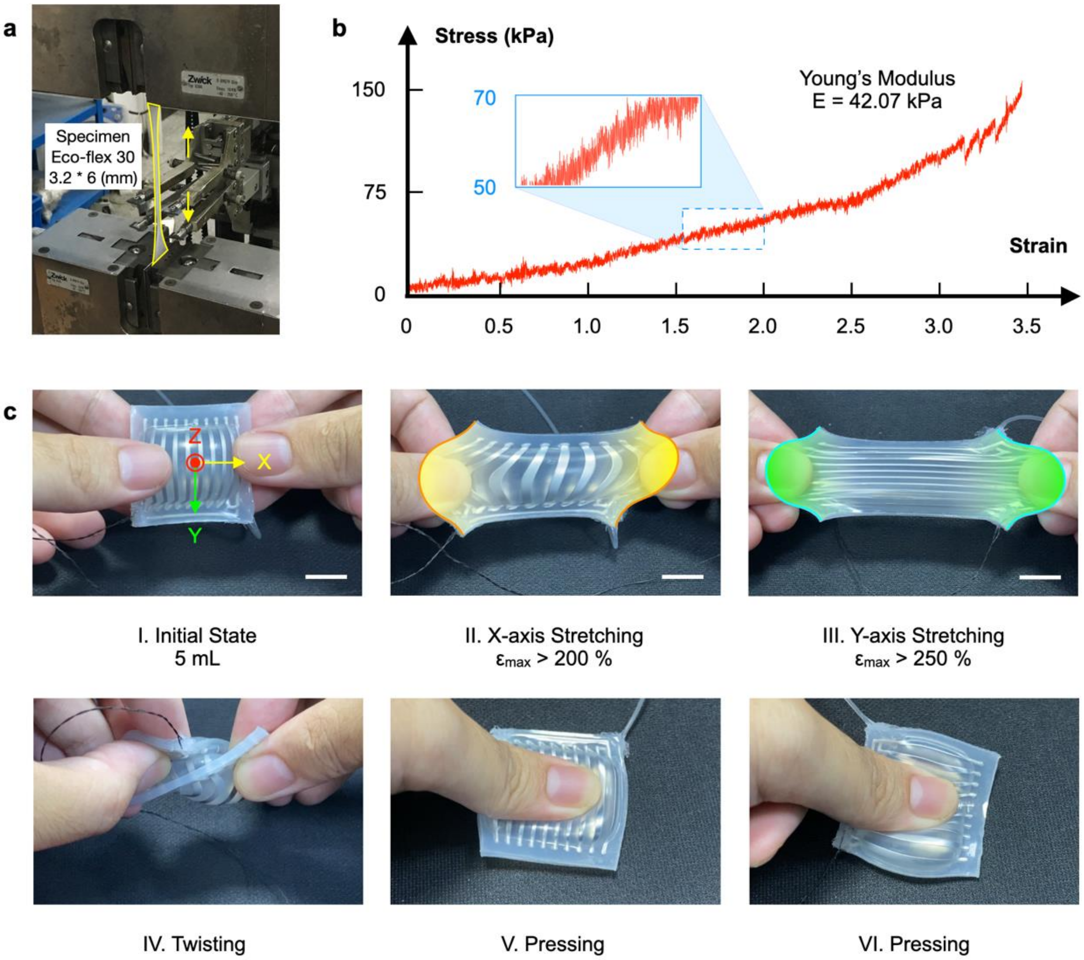

Supplementary Figure 7. Mechanical properties of Eco-flex 30 and the inflated module H.

a Tension test of the specimen made of Eco-flex 30. **b** The corresponding stress-strain curve. **c** High mechanical compliance of inflated module H. I. The air chamber is filled with 5-ml air (small amount and low pressure, as shown in Supplementary Figure 8). II. & III. X/Y-axial extensibility and its deformation (Scale bar, 10 mm). IV to VI. The robustness and deformation under other different conditions.

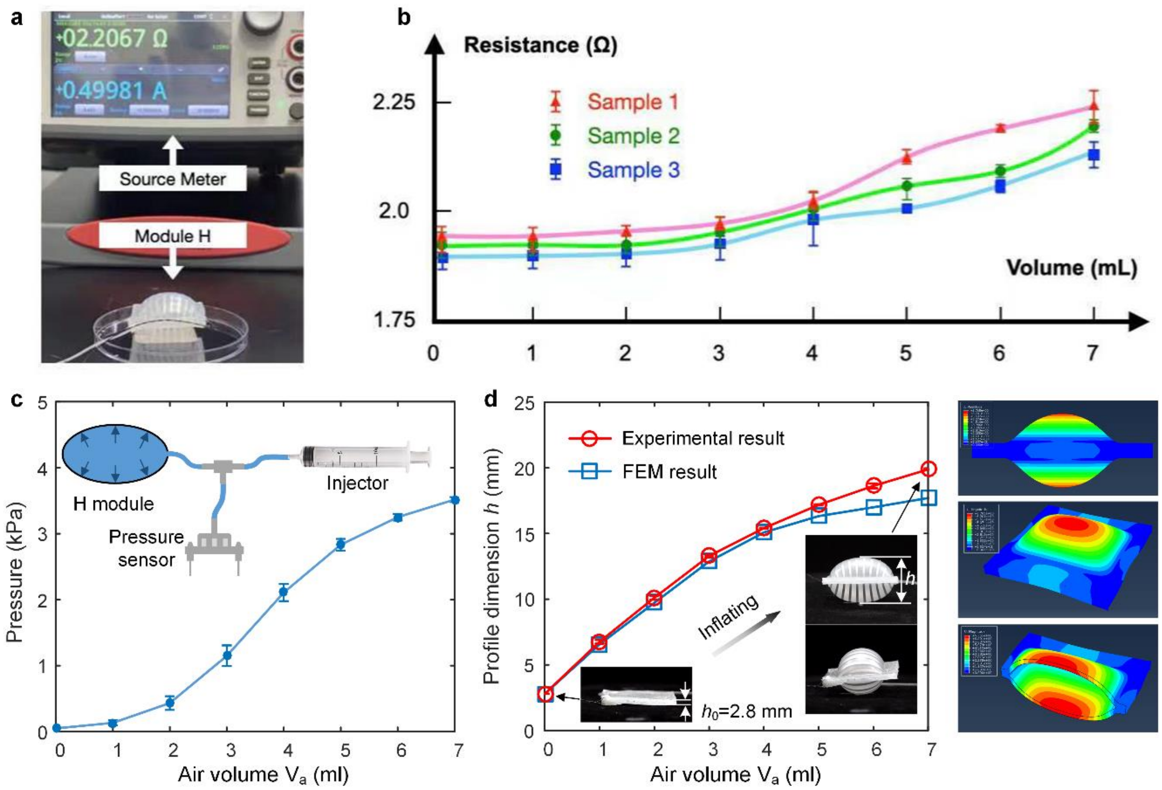

Supplementary Figure 8. Electrical, mechanical, and geometric characteristics of module H with various air volumes.

a Resistance measurement of module H. **b** Resistance changes with various air volume. The pressure of filled air on the embedded microchannels will change the resistance, and the manual assembly will give the data a slight deviation between the three samples. **c** Correspondence between internal pressure and inflation volume. The illustration shows the experimental setup, the low pressure (< 4 kPa) enables the soft actuator to maintain mechanical compliance. **d** Shape morphing and corresponding profile dimension with various air volumes. Illustrations include FEM (finite element) simulation and experimental photography from different perspectives.

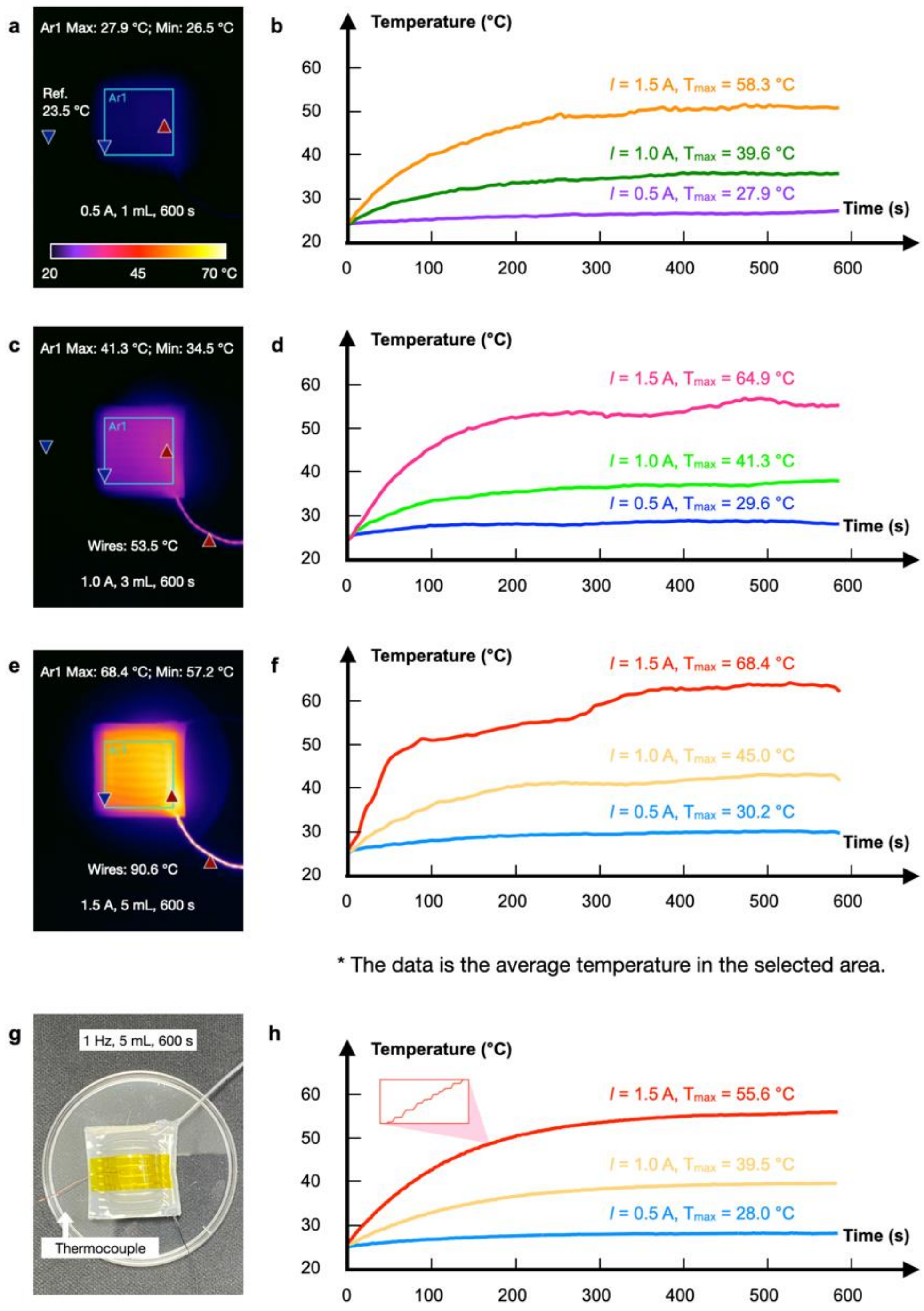

Supplementary Figure 9. Temperature measurement of module H.

a, **c**, **e** Infrared images with different air volumes and constant current. The reference room temperature is 23.5 ℃, we selected the center part of the soft actuator with a square box to display the local maximum and minimum temperature. **b**, **d**, **f** Static temperature-time curve for the soft actuator subjected to

different static currents, I = 0.5, 1.0, 1.5 A. All plotting data are from the average temperature by infrared imaging, and marked with the local maximum temperature. **g** Experimental setup for dynamic temperature test (sampling frequency = 1 Hz). The flexible thermocouple is attached to the central area. **f** Dynamic temperature-time curve and detailed local data (dynamic frequency = 1 Hz). It is worth noting that the highest temperature for invasive medical devices should not exceed 44 ℃ to avoid damaging the surrounding tissues. Therefore, for our small-scale electromagnetic robots, as long as the current is below 1 A or short actuation (1.5 A within 100 s), they can meet the requirements even as implanted devices.

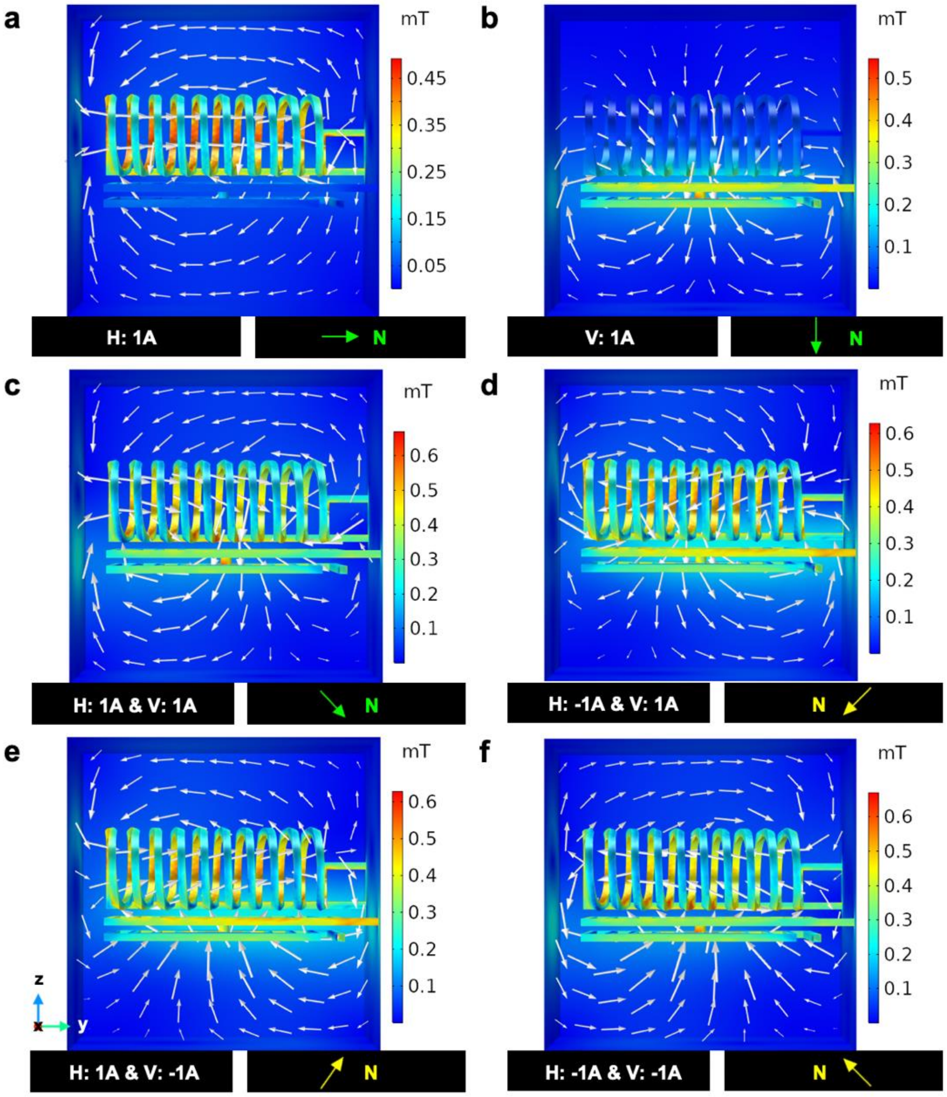

Supplementary Figure 10. Simulation of electromagnetic vector synthesis, composite module V||H.

a Separate electromagnetic vector of H module. When the input current is in the positive direction, we define the electromagnetic vector as an N pole to the right. **b** Separate electromagnetic vector of V module. Similarly, we also define the positive direction of the electromagnetic vector. **c-f** Four different synthesis results of H-V type electromagnetic vectors, by separately controlling the input currents (vectors containing reverse currents are marked in yellow). The intensities are the same. Numerically, $B_{H-V}^2 = B_H^2 + B_V^2$ (B: magnetic flux density, unit: mT).

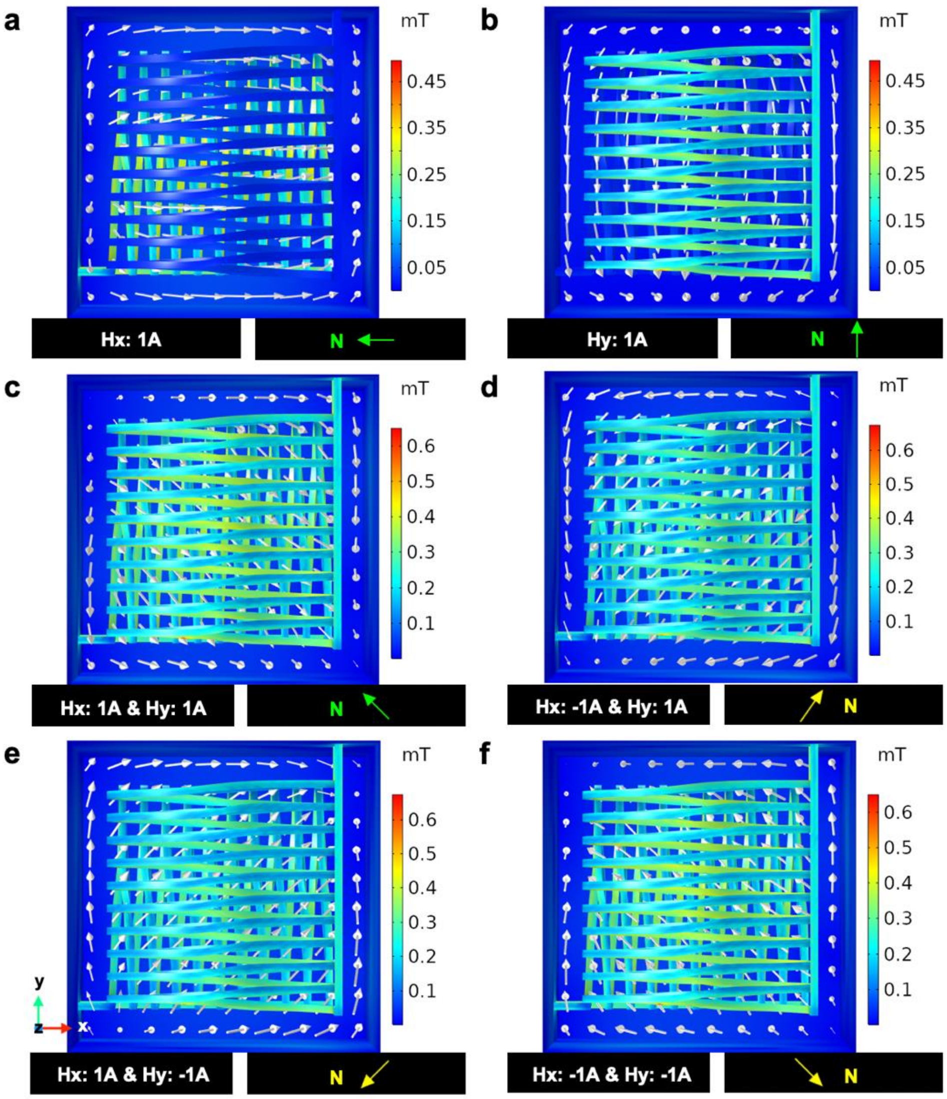

Supplementary Figure 11. Simulation of electromagnetic vector synthesis, composite module H||H.

a Separate electromagnetic vector of *H*x module. When the input current is in the positive direction, we define the electromagnetic vector as the N pole to the left (The arrow in the figure is located in the median plane of the two coils, which represents the external magnetic field of each coil. Thus, it points to the right here). **b** Separate electromagnetic vector of *H*y module. **c-f** Four different synthesis results of *H*x-*H*y type electromagnetic vectors, separately controlling the input currents (vectors containing reverse currents are marked in yellow). The intensities are the same. Numerically, $B_{Hx-Hy}^2 = B_{Hx}^2 + B_{Hy}^2$.

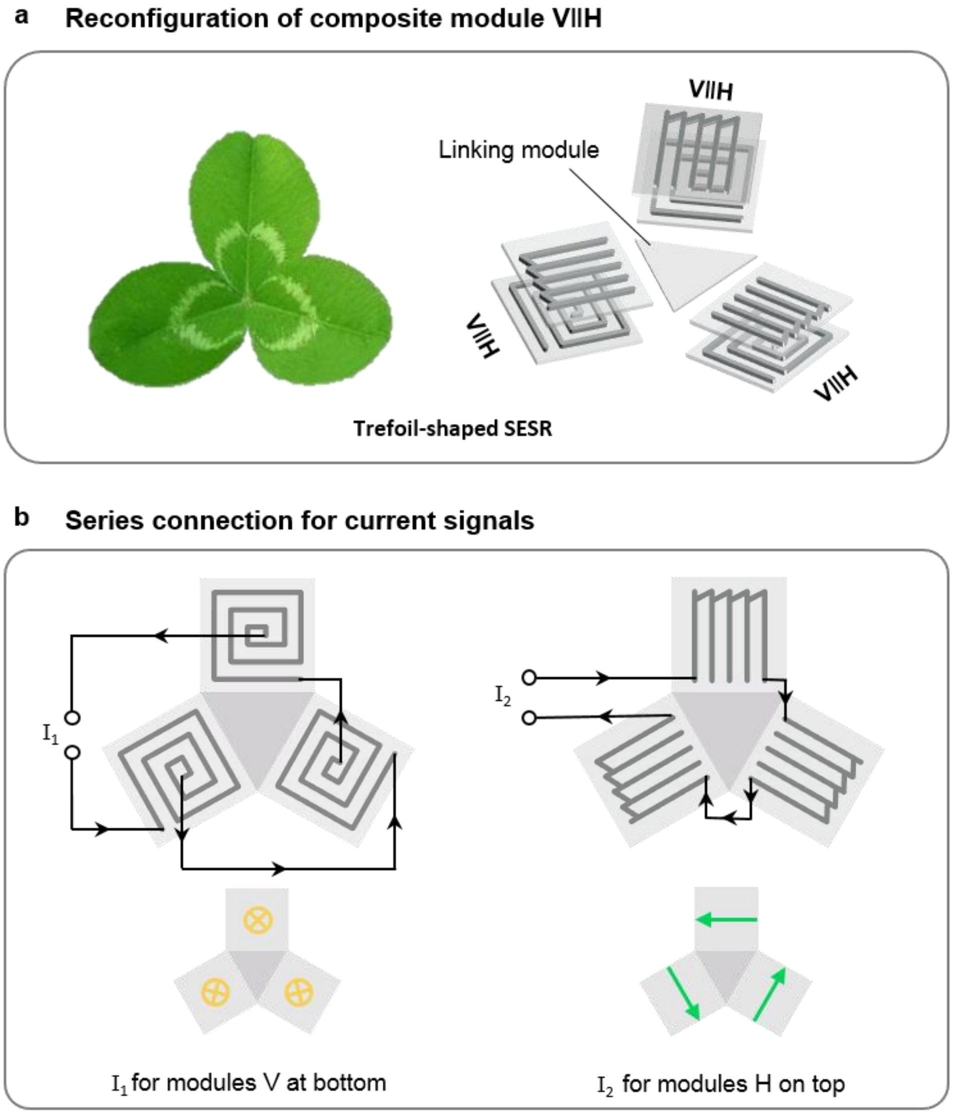

Supplementary Figure 12. The structure and control of the trefoil-shaped SESR.

a The trefoil-shaped SESR consists of three composite modules H||H and a middle triangular linking module. **b** The three V modules at bottom and the three H modules on top are connected together in series, respectively. The connection modes for series circuit shown here are mainly schematic to demonstrate the synchronous control of multiple modules by one signal. The practical connection modes are according to structures of the double-layered LM coils in module prototypes. Two current signals I_1 and I_2 are provided for the self-vectoring control.

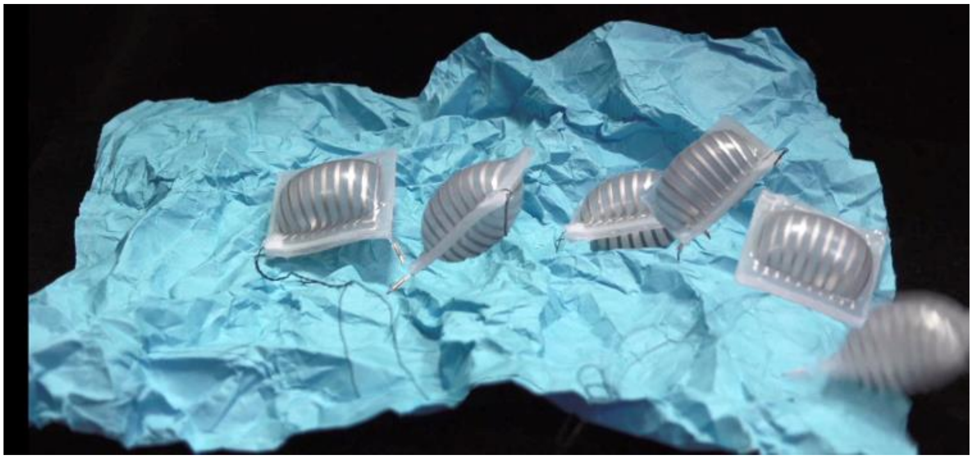

Supplementary Figure 13. Demonstration of the agile rolling on a rugged terrain simulated by crumpled paper of a single module H.

A piece of A4 paper was crumpled by hand to form a rugged surface, and the plate magnet was put below the paper to generate a magnetic field through the paper. Controlled by a single current signal, the module realized 5 flips during the continuous rolling process, demonstrating good terrain adaptability and locomotion ability.

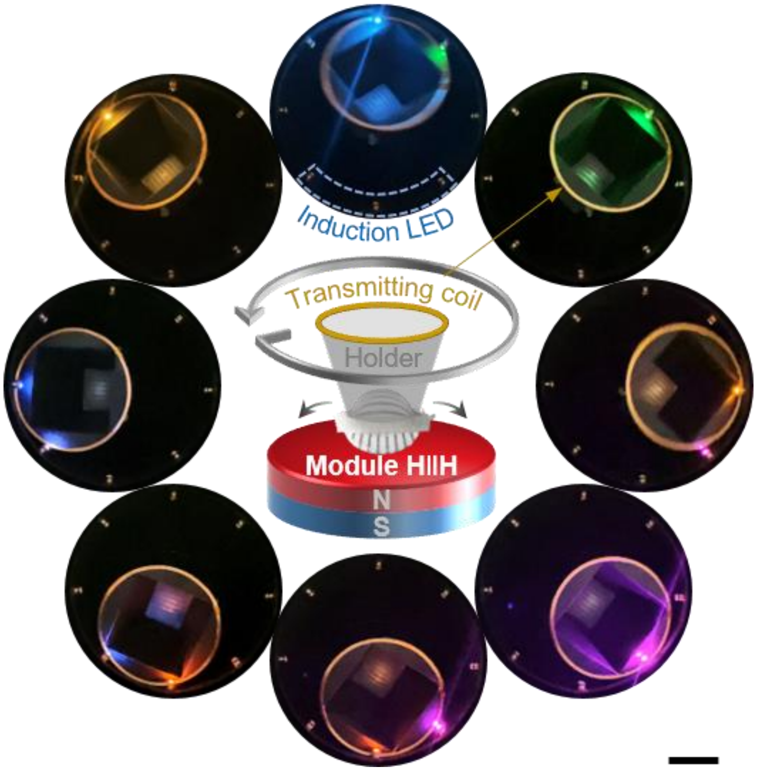

Supplementary Figure 14. Demonstration of lighting LEDs by omnidirectional rotary movement of a composite module H||H.

The inflated module H||H carries a transmitting coil (11.6 g along with the origami holder) and rotates around the center axis of the magnet, while eight colorful induction LEDs arranged evenly on the inwall of a cylinder frame are lit in sequence counterclockwise (Supplementary Movie 2). Two current signals connected to the two module H actuators can generate eight EVs corresponding to the directions of eight LEDs. EVs are synthesized and switched around a plane parallel to the magnet surface, which rotates the composite module to light the LEDs in sequence. Scale bar, 30 mm.

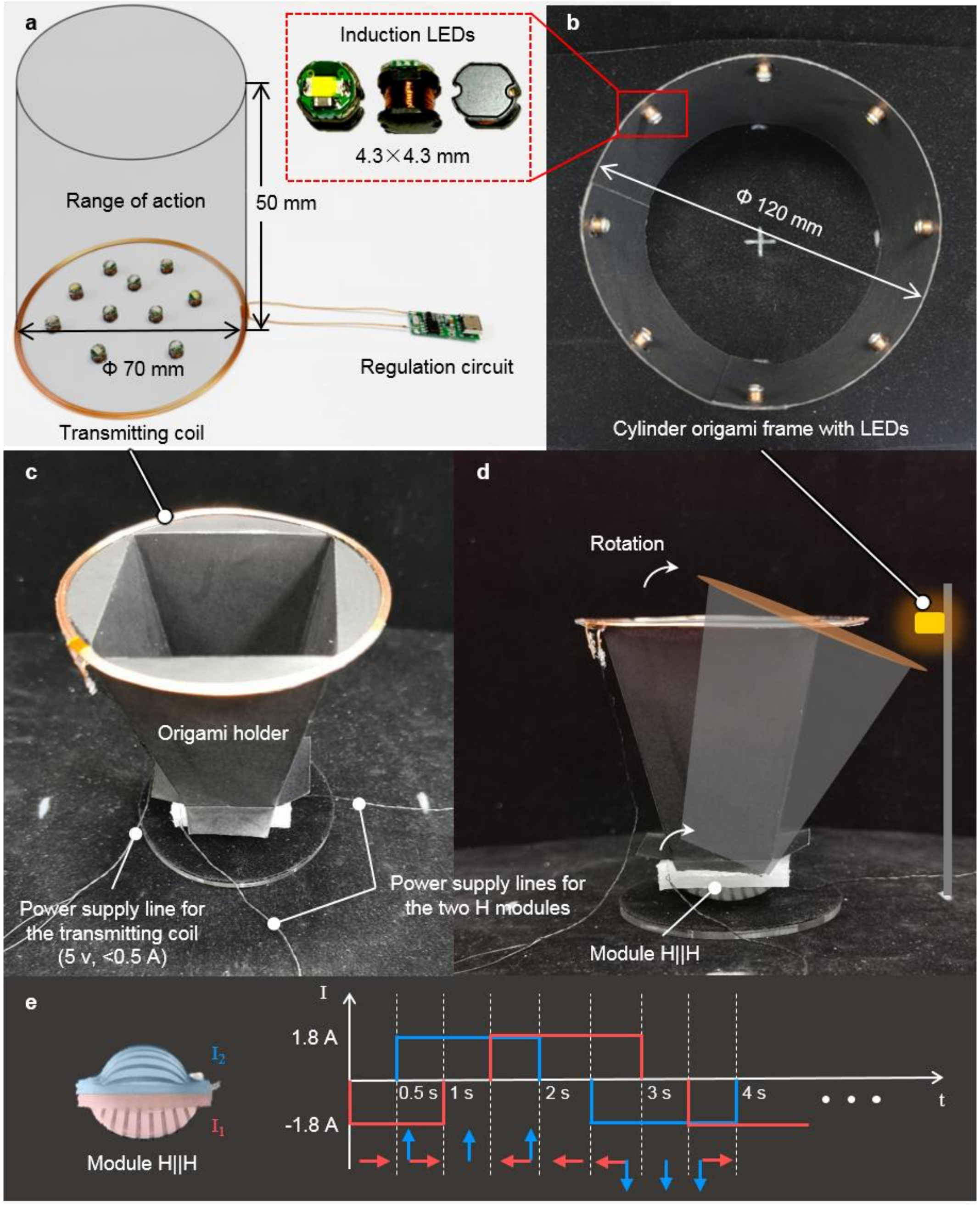

Supplementary Figure 15. Experimental setup and control signal for the rotary lighting demonstration of composite module H||H.

a The wireless transmitting coil and several induction LEDs (JH56D, ADA, China). The transmitting coil is connected with a USB interface and can be powered by a computer. The action range of the transmitting coil is a cylindrical region with 70-mm diameter and 50-mm height. The induction LEDs consists of LEDs with different emitting colors and the tiny receiving coil circuit. **b** A homemade cylinder

origami frame for arranging induction LEDs. The frame with 120-mm inner diameter and 85-mm height was made from black card papers (230-250 g m^{-2}, Qurui, Zhejaing, China). Eight colorful induction LEDs were mounted around the inwall of the frame evenly by double-sided tape (Deli, China). The distant from the LEDs to the magnet surface is 75 mm. **c** The homemade origami holder connecting the bottom module H||H and the top transmitting coil. The configuration of the holder is like a trumpet, with narrower opening for connecting the module and the broader one for carrying the transmitting coil. It was also made from the black paper with final height of 60 mm. **d** The lighting process controlled by the rotation movement of the bottom module H||H. Tilting of the transmitting coil can bring the induction LEDs into the action range of electromagnetic induction, thus lighting the LEDs. **e** The current pattern for the two elementary H modules to realize the omnidirectional rotary movement on the magnet. Rotation of the transmitting coil carried by module H||H can light the circumambient induction LEDs in sequence.

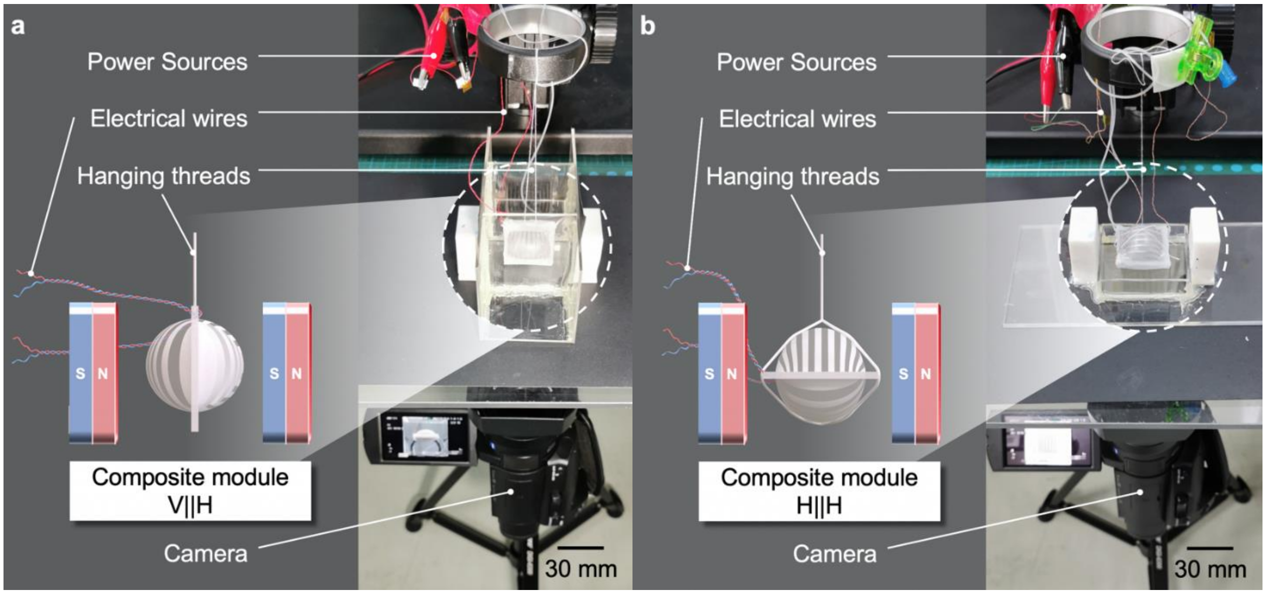

Supplementary Figure 16. Experimental setup for validating the orientation control or self-vectoring control.

Different composite modules are suspended by cotton threads, which will rotate in the external magnetic field. Two opposite magnets with different magnetic poles are fixed on the transparent shell to form a nearly parallel magnetic field. The camera looks up through a piece of black paper with a square window and records the real-time movements under self-vectoring control. a Composite module V||H. b Composite module H||H.

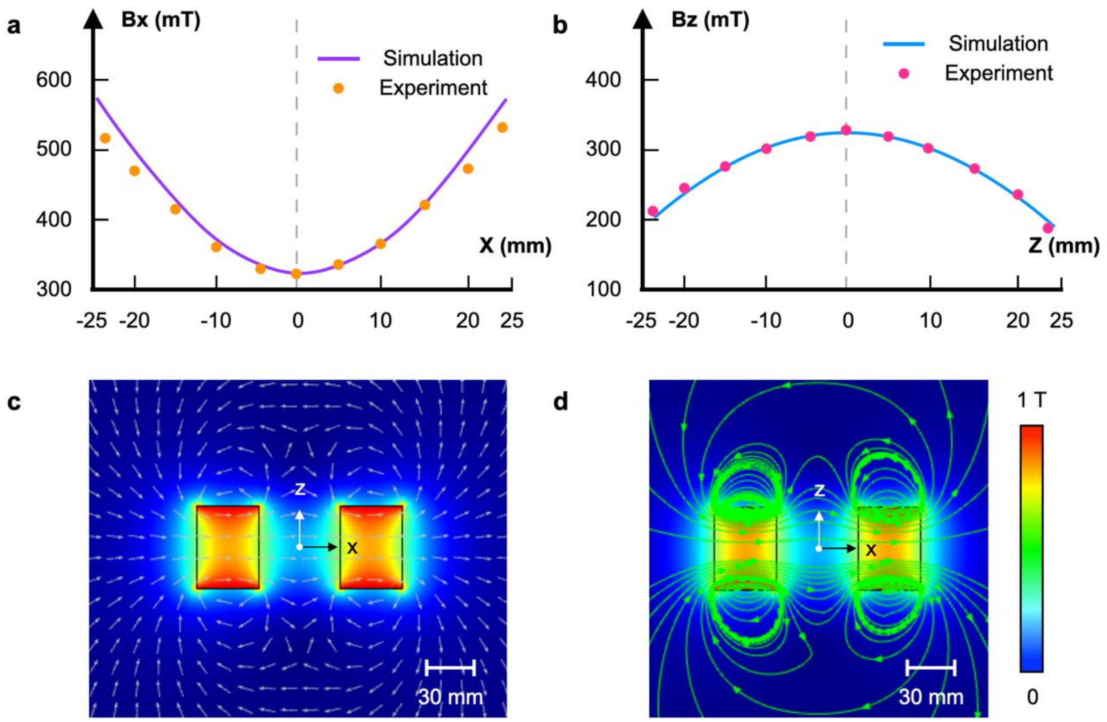

Supplementary Figure 17. Characterization of the magnetic fields produced by two opposite blocky magnets.

a Quantitative diagram of the horizontal component of magnetic flux density (*Bx*) varying with distance (*x*, from -25 to 25 mm, 0 represents the midpoint between two magnets). **b** Quantitative diagram of the vertical component of magnetic flux density (*Bz*) varying with distance (*z*, from -25 to 25 mm, 0 represents the midpoint of the central line). **c** Simulated results of the distribution of local magnetic field between two permanent magnets. **d** Simulated results of the magnetic induction line of the middle section.

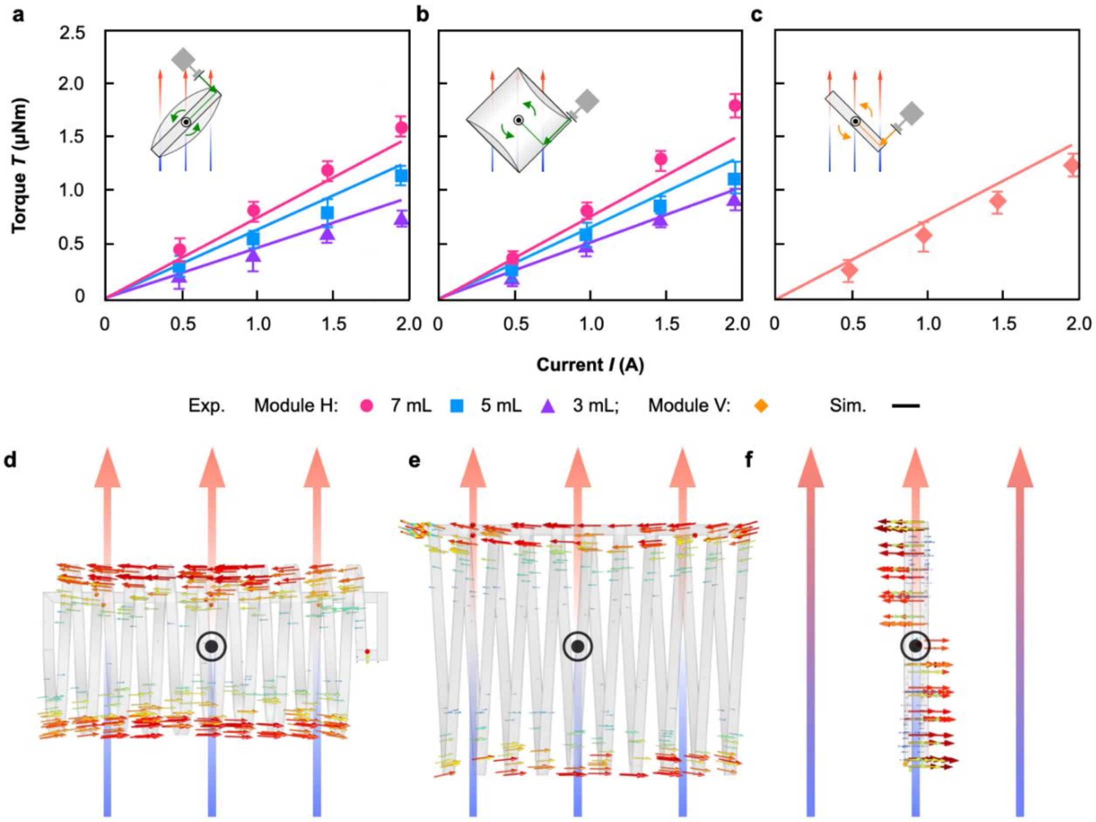

Supplementary Figure 18. Experiment and simulation of the Lorentz torque of moudles H and V.

a to **c** Comparison of experimental and simulated Lorentz torque, The EVs of modules H and V are at a 45-degree angle to the passive and constant magnetic field. Two attitudes of module H are involved. **d** to **f** Finite element simulation of Lorentz force-based moment. The black dot represents the rotation axis, and red means the maximum strength in different currents (0 ~ 2 A) for all vector arrows.

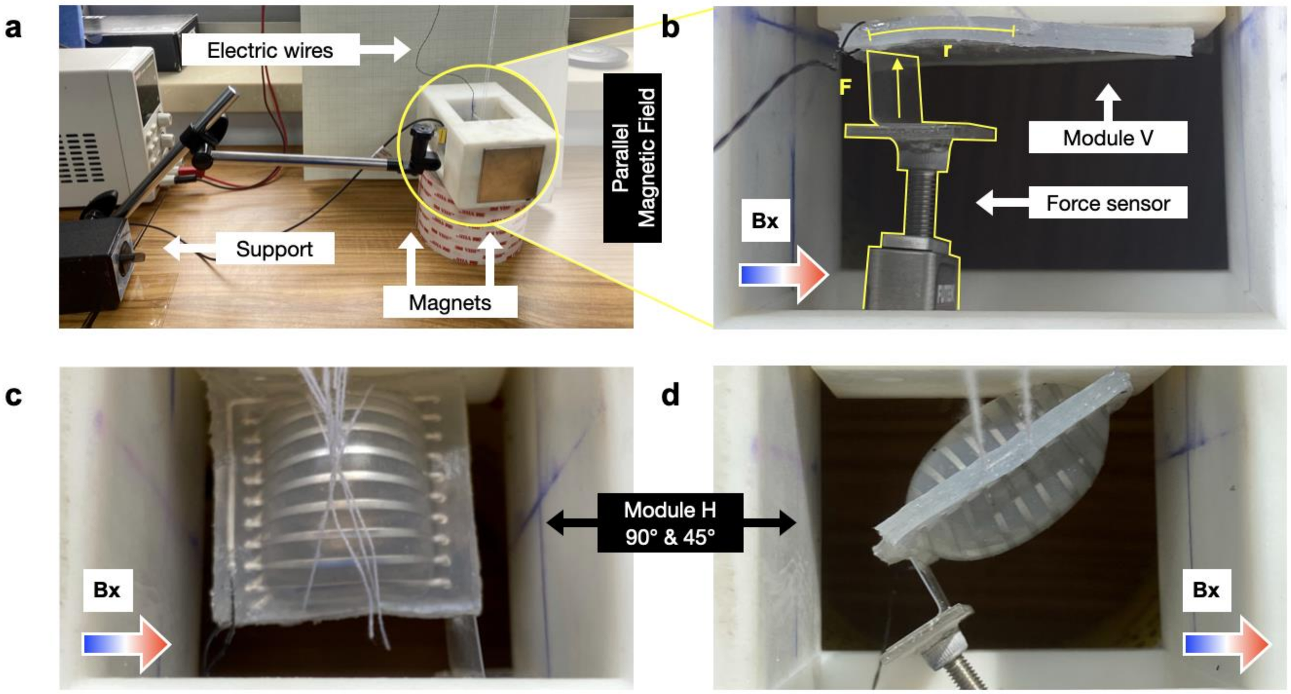

Supplementary Figure 19. Characterization of the Lorentz torque.

a Experimental setup for the torque tests. **b**, **c**, **d** Zoom-in details about module V, module H 90°, and module H 45°. The module H was suspended flat or sideways in the magnetic field corresponding to **c** and **d.** The gravity direction is perpendicular to the picture and pointing inside. Two opposite magnets with different magnetic poles are fixed on the 3D-printed fixture. All soft actuators are suspended to minimize the effects of gravity, friction, etc.

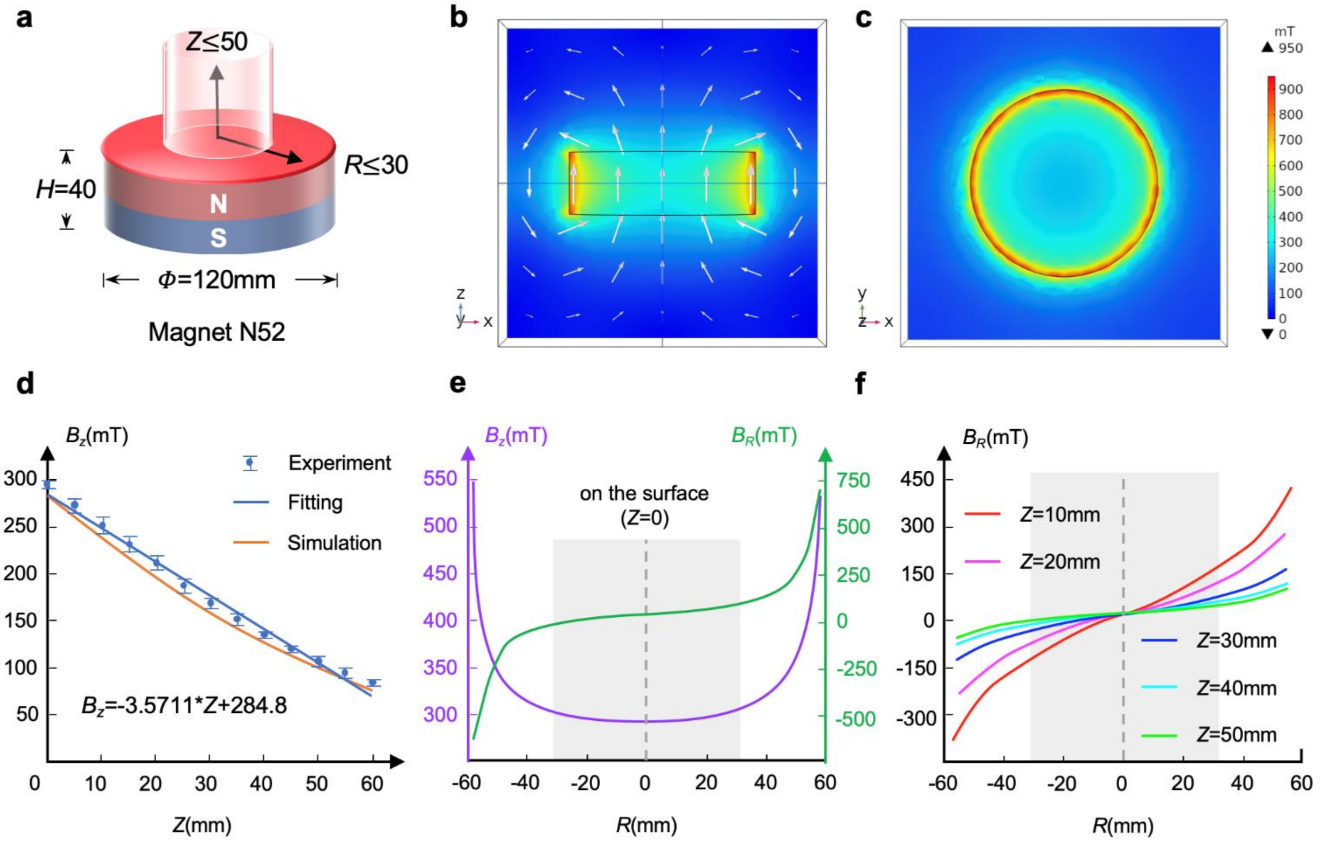

Supplementary Figure 20. Characterization of the plate permanent magnet and the actuation range of SESR.

a Schematic diagram of the plate magnet and its linear interval. **b** Simulation of magnetic field distribution and corresponding magnetic vector direction. **c** Simulation of the surface magnetic field, the legend shows the strength of magnetic flux density (*B*, unit mT). **d** Quantitative diagram of the vertical component of magnetic flux density (B_z) varying with height (*Z*, unit mm). By comparing simulation and experimental fitting, the linear function of B_z can be obtained. B_z=-3.5711**Z* + 284.8. **e** Distribution of the two magnetic flux density components on the surface of magnet N52 (B_z, purple curve & B_R, green curve). Both maintain high linearity in the range of -30~30 mm radius, and there is severe strengthening at the edge of the magnet. **f** Quantitative diagram of the horizontal component of magnetic flux density (B_R) varying with height (*Z*, from 10 to 50 mm).

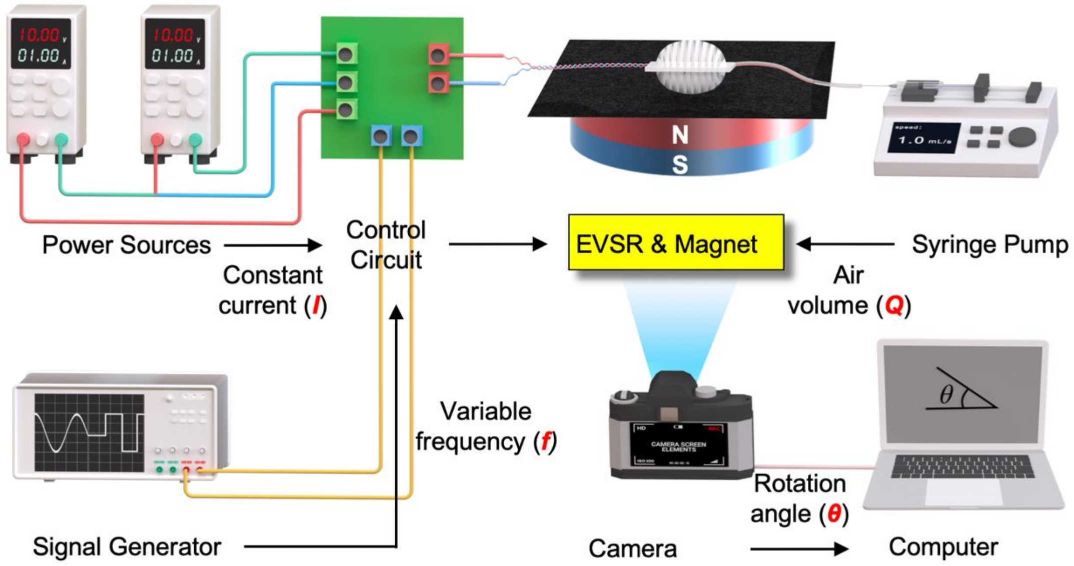

Supplementary Figure 21. Experimental setup and method for charactering actuators.

The whole setup can be divided into three parts: electronic system (power sources, a signal generator, and a control circuit), robotics system (an SESR, a magnet, and a syringe pump), motion capture system (a camera and a computer). For the robotics system (constant magnetic field), controllable input variables include: current amplitude, direction, frequency, and inflation; the final outputs are: rotation angle and speed.

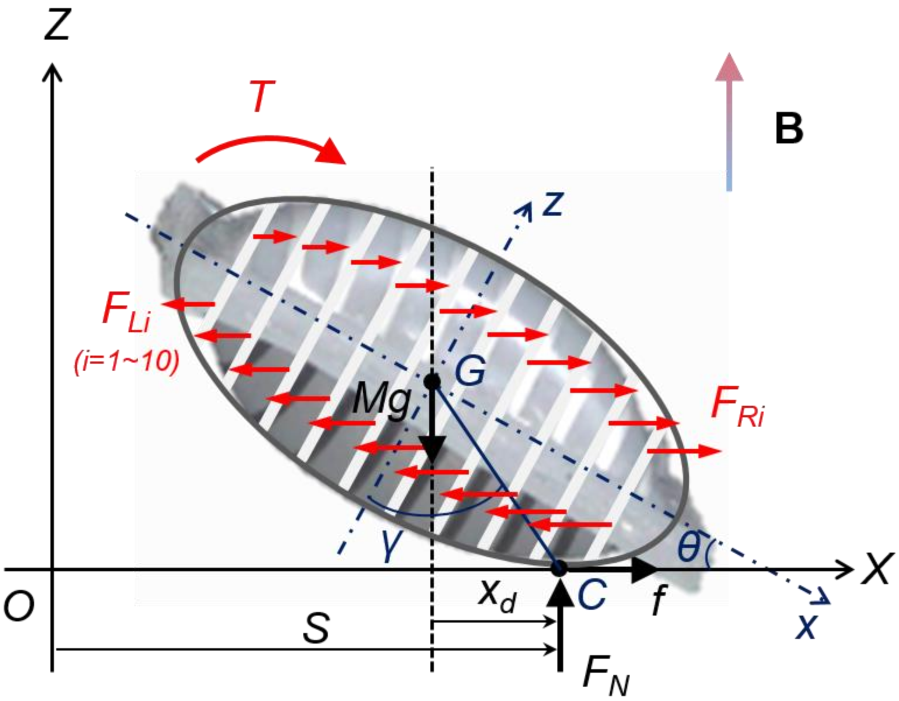

Supplementary Figure 22. The force analysis for module H rotating in a constant magnetic field.

When a DC I is applied to the module, the flowing current in the helical coil generates Lorentz force under the external magnetic field **B**. The force can be derived by $\boldsymbol{L}=\boldsymbol{I}\oint dl\times\mathbf{B}$, where dl is an infinitesimal length of the coil. According to the right-hand screw rule, we can determine the directions of the forces exerted on each circle of the coil. It shows that the upper half coil is subject to the right Lorentz force F_{Ri} (i represents the ith circle of the LM coil), which can induce a clockwise torque T with respect to the contact point C between the module and the surface. While, the bottom half coil is subject to the left Lorentz force F_{Li}. Equilibrium under the combined action of F_{Ri}, F_{Li}, Mg (gravitational force), F_N (reaction force), and f (frictional force), the module finally stabilizes on the magnet with a tilt angle of θ. Signal with dynamic loading could also lead to dynamic rotation motion for the module.

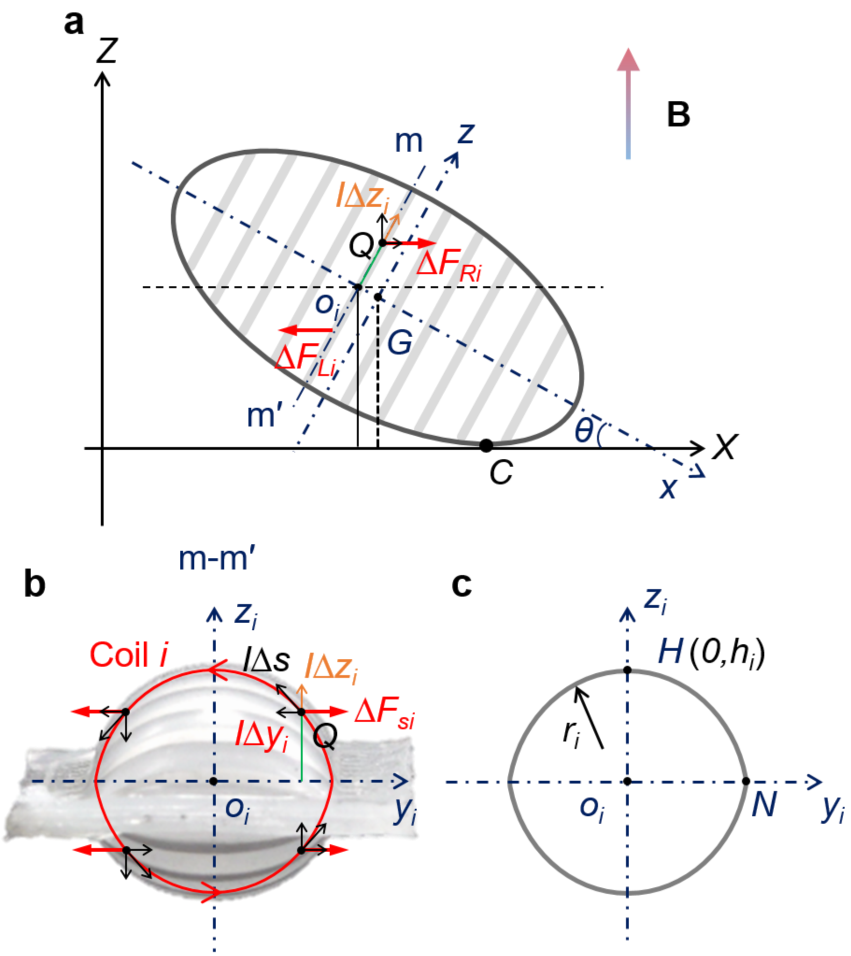

Supplementary Figure 23. Analysis of the components of the Lorentz force exerted on the coil.

a The abstract model of the module H for analyzing the rotation motion on the surface. **b** The cross-section of the module and the Lorentz force on the *i*th coil. **c** The geometry model of the coil cross-section.

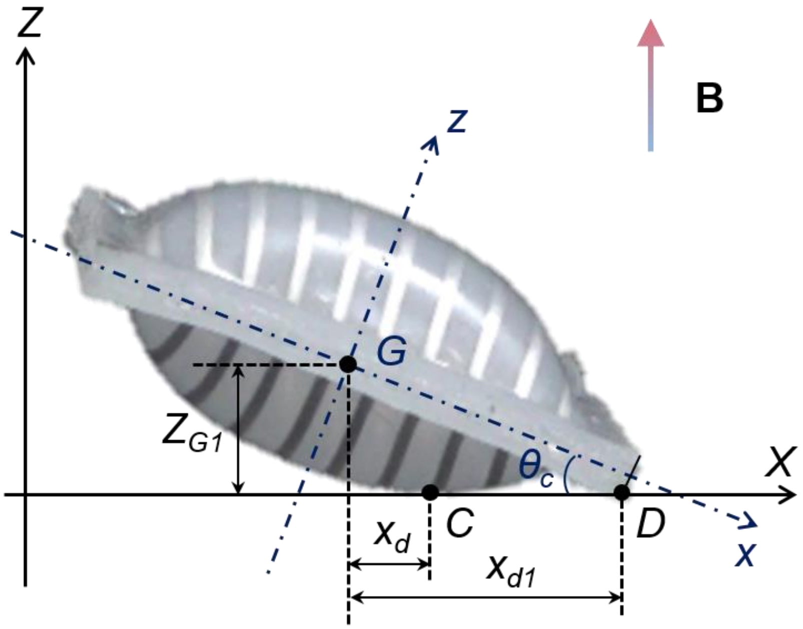

Supplementary Figure 24. The transition state for different rotation tendencies of module H.

At this critical state, the endpoint of the convex part of the practical module H begins to contact the surface at point *D*. So the rotation center changes from point *C* to point *D* with the increasing of the current.

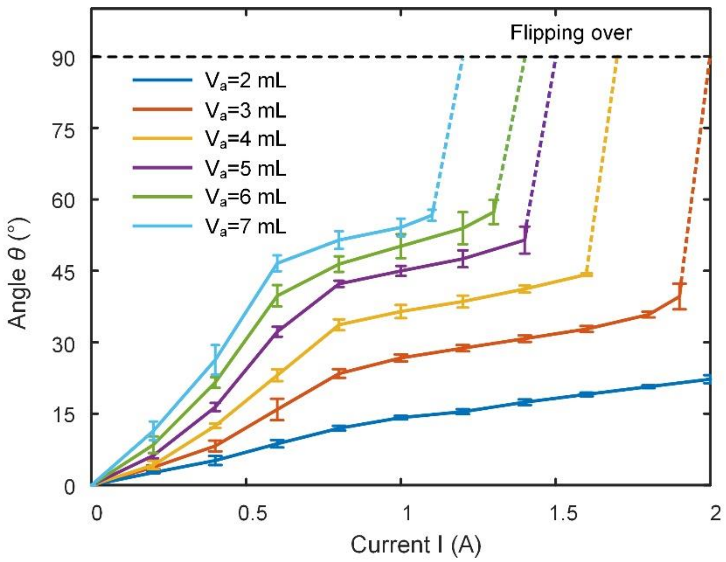

Supplementary Figure 25. Experimental results of the steady rotation angle of module H with different inflating volumes varying with the applied current.

The angle first increases with the current, then gradually tends to gentle. When the current reaches a critical value, mdodule suddenly flips over. A larger inflating volume corresponds to larger steady angle under the same current, leading to a samller critical current for flipping. When the current increases slowly from 0 to 2 A, we didn't observe the flip motion for the module with 2-ml air volume. The critical currents measured experimentally for V_a=3-7 ml are 1.9A, 1.6A, 1.4A, 1.3A, and 1.1A, respectively. The comparision of theoretical and experimental critical currents can be seen in Supplementary Table 3.

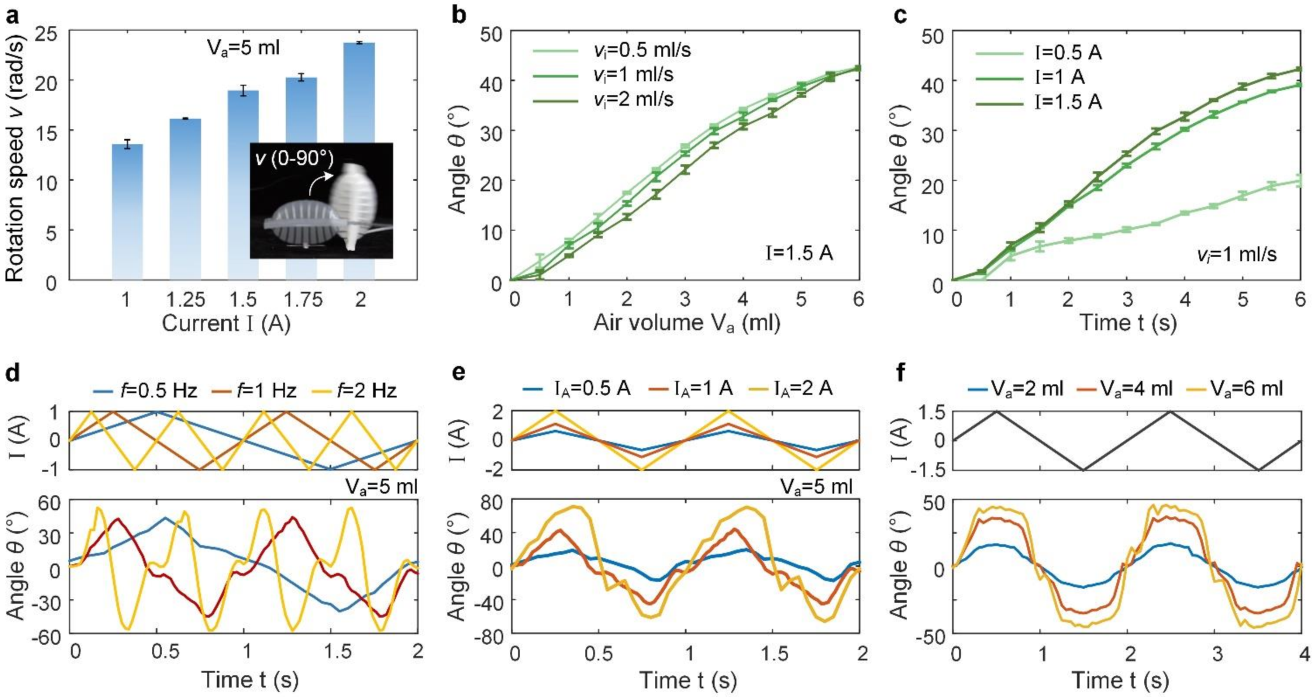

Supplementary Figure 26. Actuation characterization of module H on a plate magnet.

a The average rotation speed *v* varying with the current for the module with V_a=5 ml when flipping over 90 ° (step signals applied). **b** The rotation angle varying with air volume V_a for the module with three different inflating rates v_i (a 1.5-A constant current). **c** The rotation angle varying with time for the module when inflated by a rate of 1 ml s^{-1} and under different excitation currents. **d** The periodic response of rotation angle for the module when subject to AC triangular-wave current signals with different frequencies and same1-A amplitude. **e** The periodic response of rotation angle for the module when subject to AC triangular-wave current signals with different amplitudes, *f*= 1Hz. **f** The periodic response of rotation angle for the module with varying air volumes when subject to an AC triangular-wave current signal with 1.5-A amplitude and 0.5-Hz frequency.

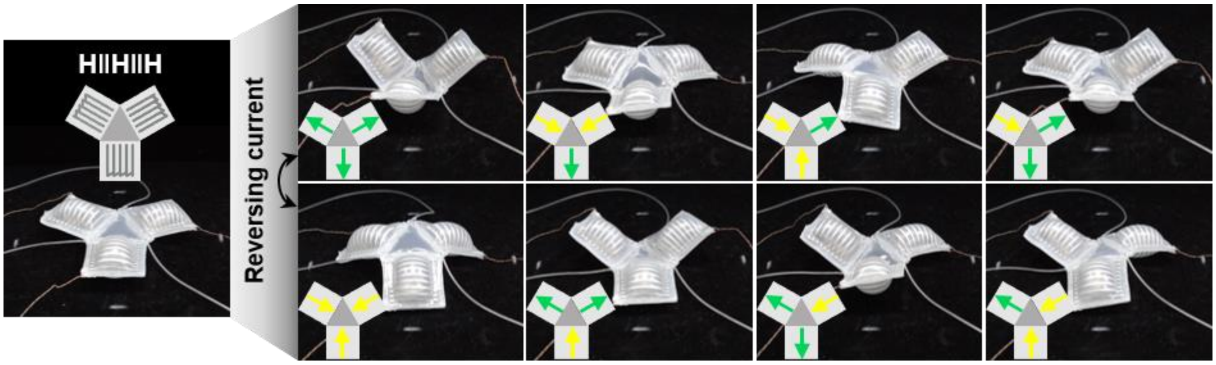

Supplementary Figure 27. Reprogrammable shape morphing of an H||H||H robot.

Under a constant unidirectional magnetic field, by controlling each H module independently or synchronously, the trefoil-shaped SESR can perform eight different fast reprogrammable transformations.

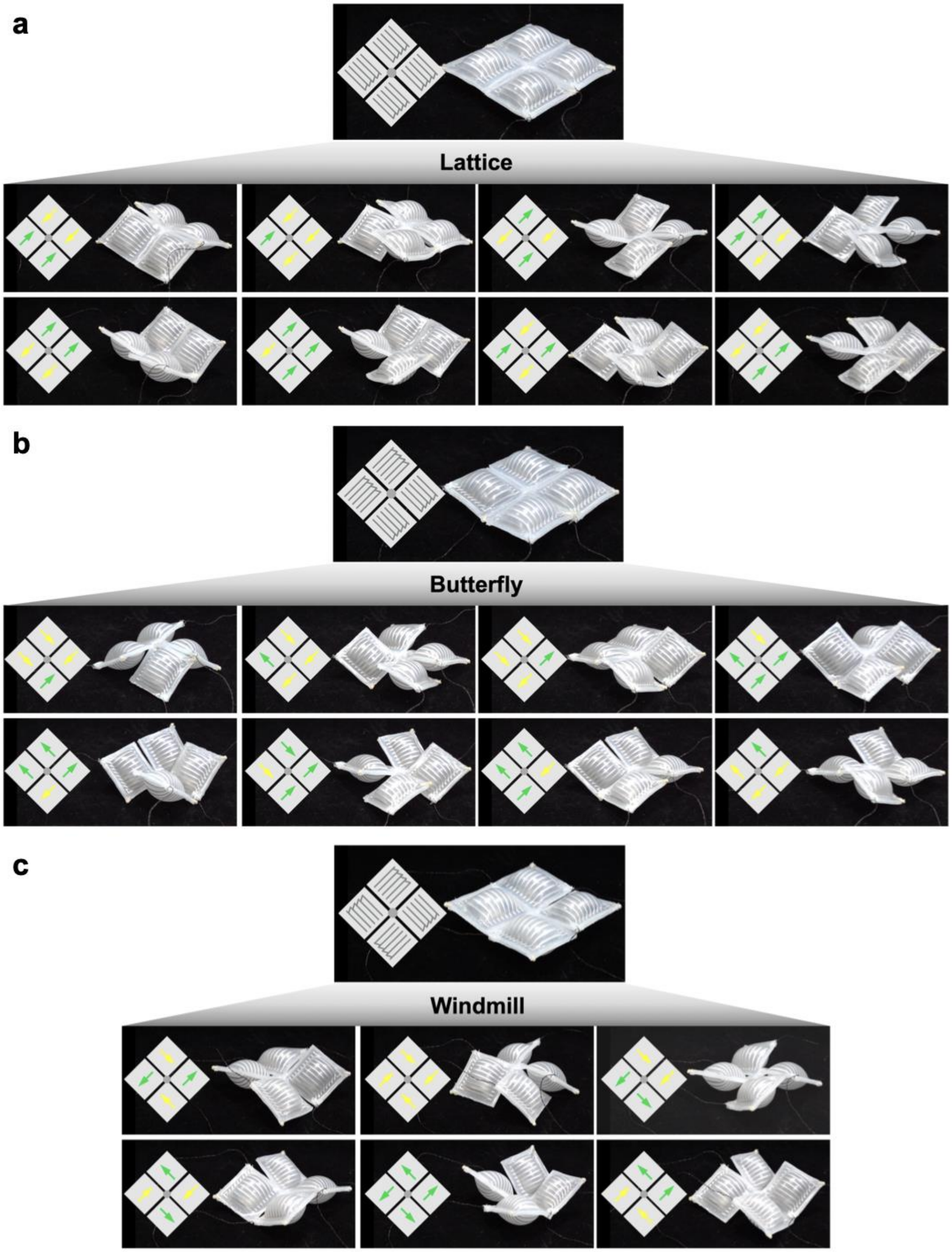

Supplementary Figure 28. Reprogrammable shape morphing of an H||H||H||H robot.

Three typical square structures reconfigured by four H modules with different orientations **a** Lattice form, **b** Butterfly form, **c** Windmill form, and their fast reprogrammable transformations.

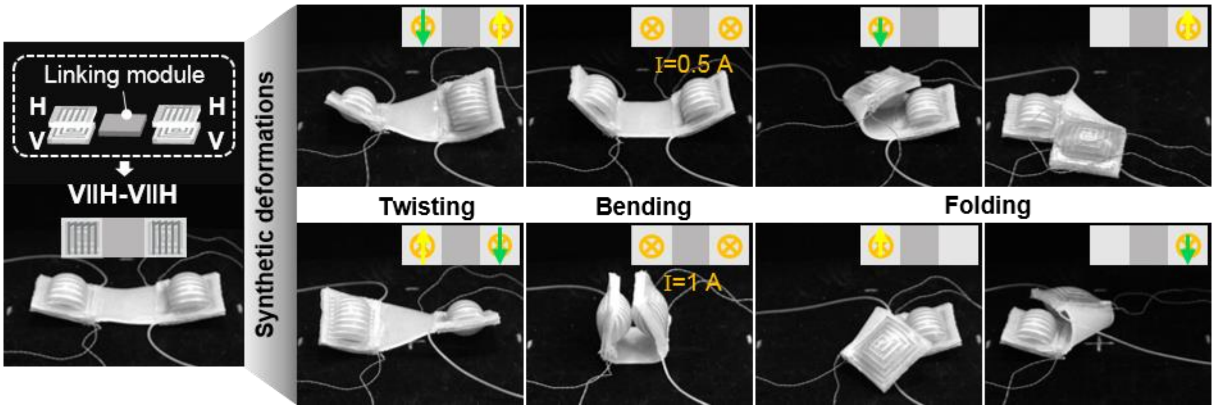

Supplementary Figure 29. Reprogrammable shape morphing of a V||H-V||H composite robot.

The strip structure obtains more operational dimensionality and transformations by connecting the two V||H composite modules with a linking module (soft elastomer matrix made by Eco-flex 30). (i) Twisting: two H modules are controlled simultaneously to generate horizontal electromagnetic vectors in opposite directions. (ii) Bending: two V modules are controlled simultaneously to generate vertical electromagnetic vectors. The larger the current, the stronger the repulsive force with the magnet. (iii) Folding: When the V||H composite module on one side is powered, the SESR can be folded like origami.

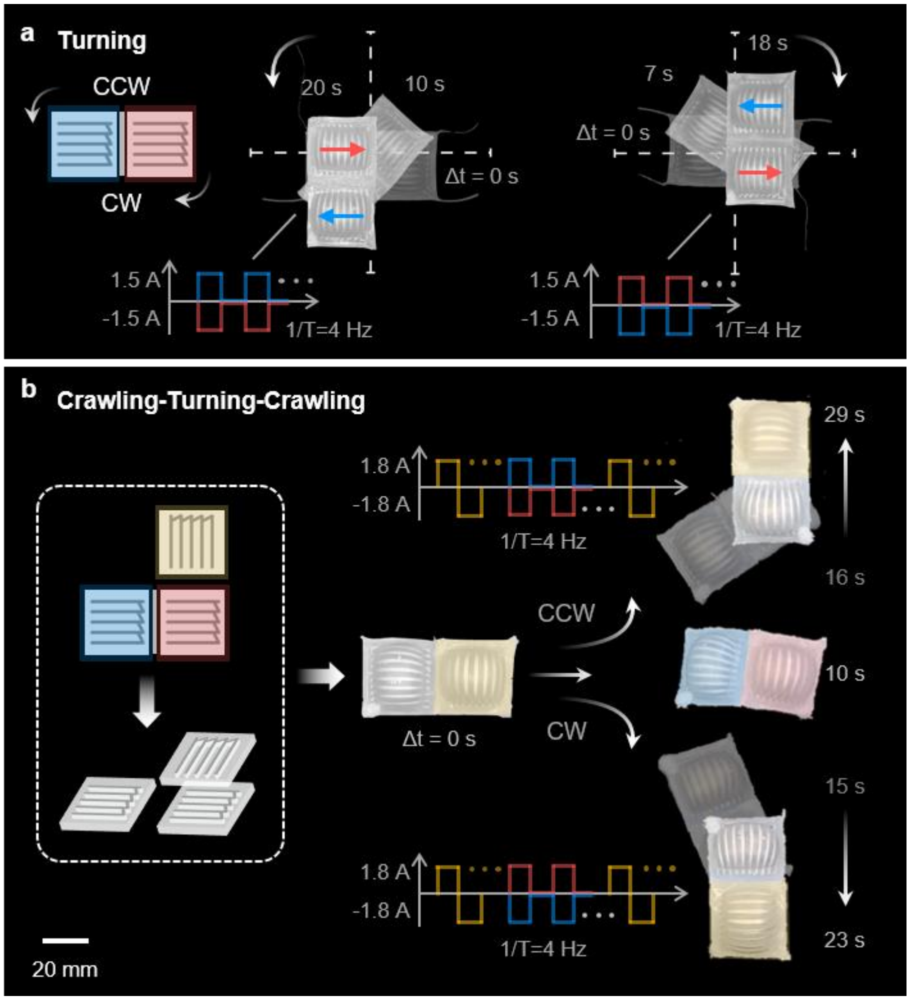

Supplementary Figure 30. Turning and crawling locomotion of the SESRs.

a Turning locomotion of an H-H robot with parallel EVs. Applying square-wave signals with opposite polarity on the two H modules can lead to clockwise and counterclockwise turning locomotion. **b** Crawling and turning of an H-H||H robot. It can be seen this robot is the combination of the two foregoing H-H robots with crawling and turning locomotion capacities respectively. When crawling, the upper H module oscillates periodically and acts as a traction head to drive the whole robot to crawl forward. While for turning, the bottom two serial H modules are activated with opposite currents, the modules then sway to drive the robot turning clockwise or counterclockwise under corresponding control signals.

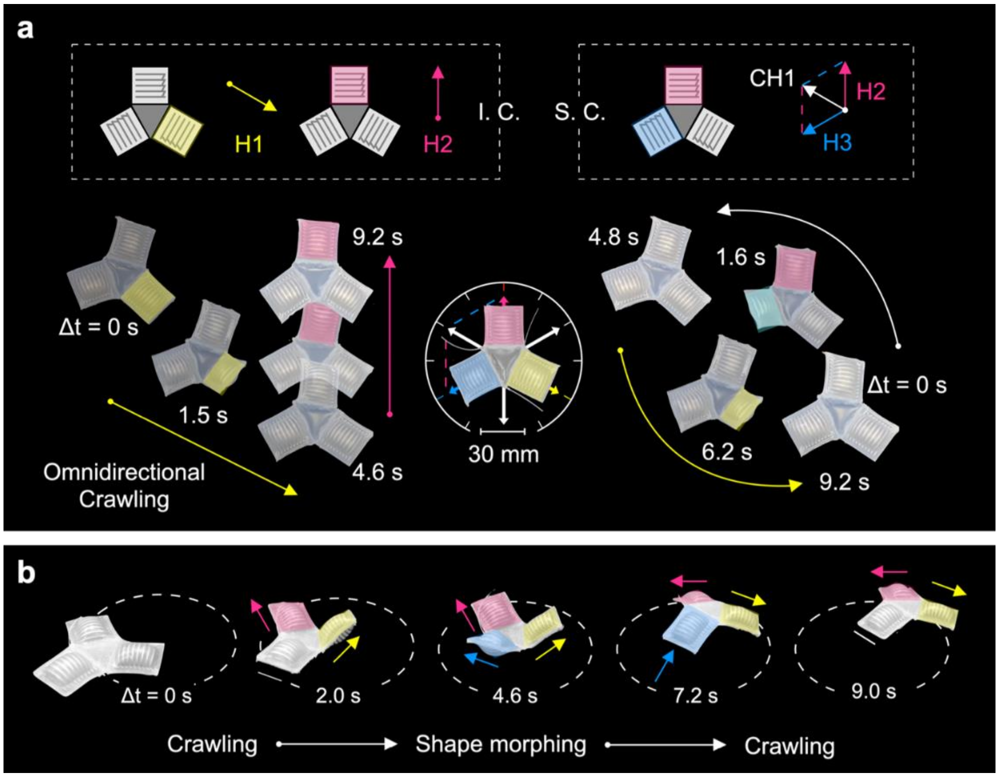

Supplementary Figure 31. Omnidirectional crawling of an H||H||H robot.

a Two representative crawling modes with the corresponding electromagnetic vectoring. Independent control (I. C.): By controlling an actuator separately, the robot can crawl in its direction (we powered the yellow module firstly); Changing the driving module means changing the crawling direction (then the red module). Synchronization control (S. C.): Based on the synthesized electromagnetic vector (the red and blue modules act as components simultaneously), the robot can perform more crawling directions. **b** Multi-mode locomotion of the H||H||H robot. The robot can complete dynamic deformation by supplying power to an actuator that did not provide an electromagnetic vector during crawling.

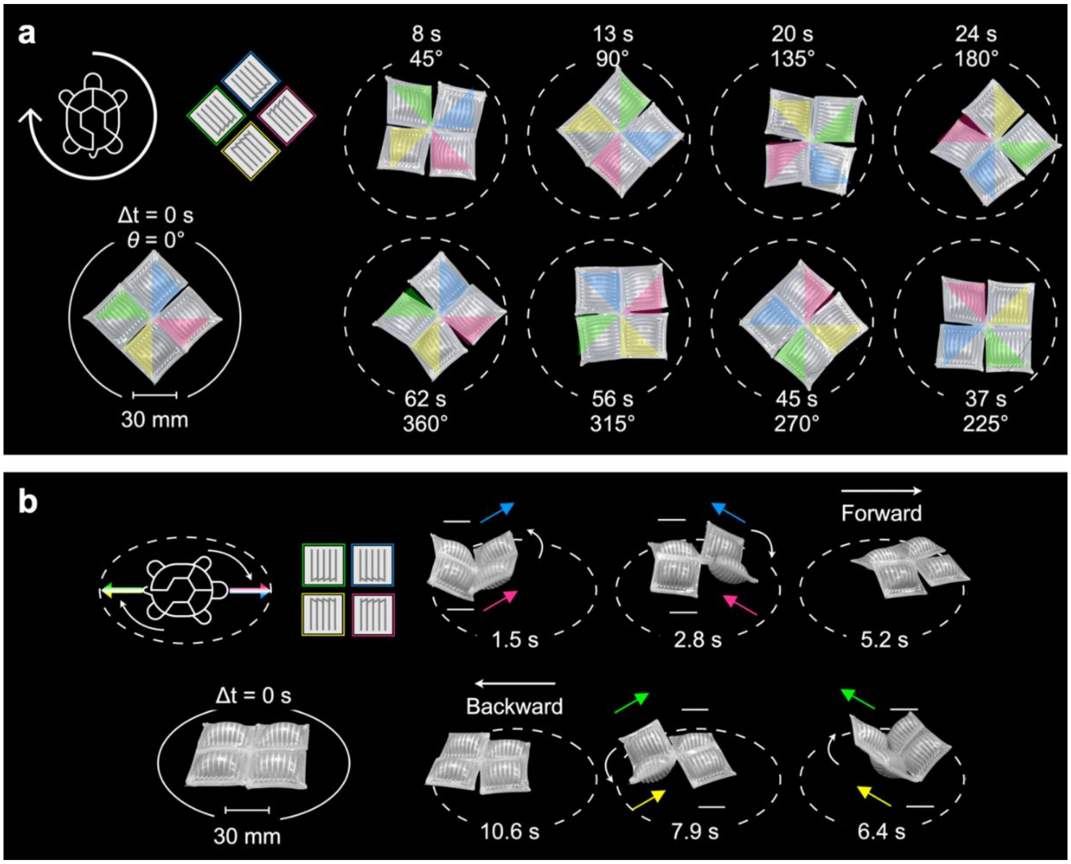

Supplementary Figure 32. Multi-mode locomotion of an H||H||H||H robot.

a Rotating in circles with a speed of about 5.8 ° s^{-1}. Bioinspired by the tortoise, when two groups of actuators on the diagonal generate reverse electromagnetic vectors, the robot can demonstrate fast rotation (>360 °) under the control of an oscillating square wave signal. **b** Reciprocating crawling with a speed of about 12 mm s^{-1}. When a group of two adjacent actuators generate the same electromagnetic vector, the robot crawls in the corresponding direction.

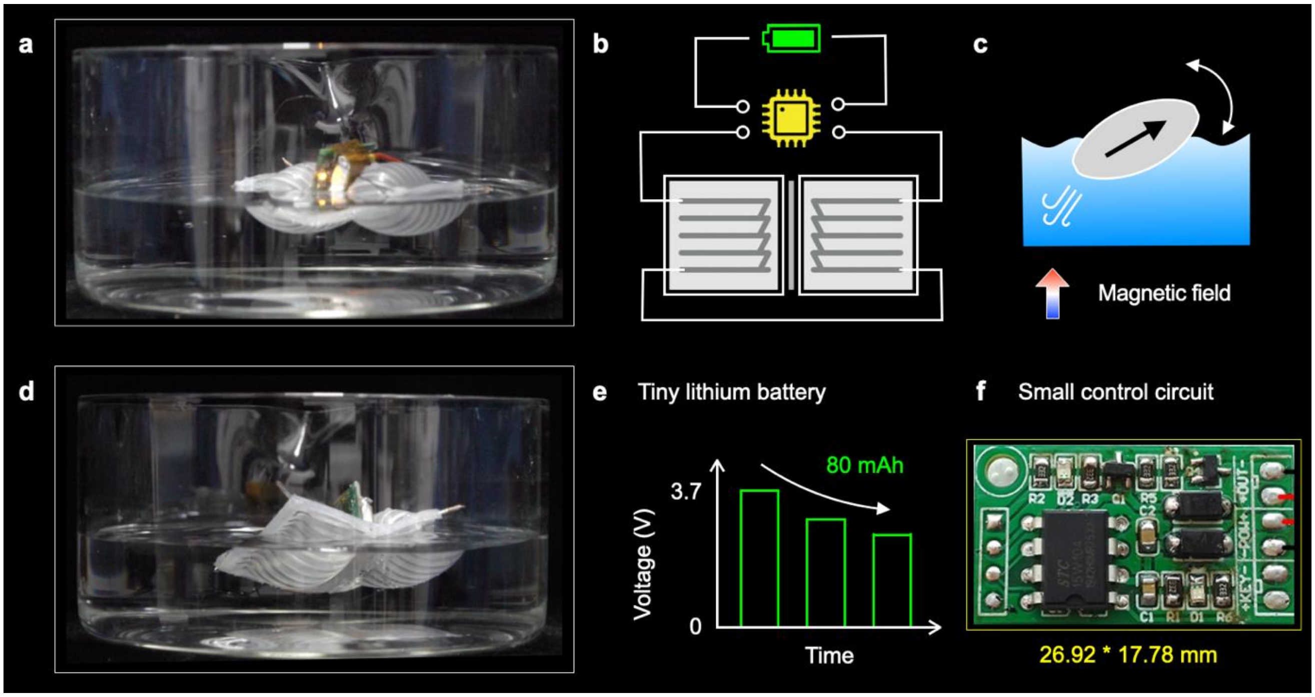

Supplementary Figure 33. Untethered SESR integrated with power and control circuit.

a Untethered SESR floating on water in standby mode. **b** Architecture of the integrated system, including a miniature polymer lithium cell, a small PWM control circuit, and two modules H. **c** Actuation and paddling principles on the water. The control circuit is a commercial time delay module enabled by MOSFET, which can generate PWM control signals with tunable frequency and duty cycle. A signal with an amplitude of 3.7 V and frequency of 4 Hz was set to control the untethered SESR to paddle on the water and further swim on the surface. **d** Untethered SESR partially immersing in water in actuation mode. **e** Schematic of the damped output powered by the 3.7-V polymer lithium cell with an 80 mAh capacity. **f** Picture of the commercial time delay module. SESR was connected to the terminals "+OUT-", the battery was connected to "-POW+"; "+KEY -" connected to the on-off switch.

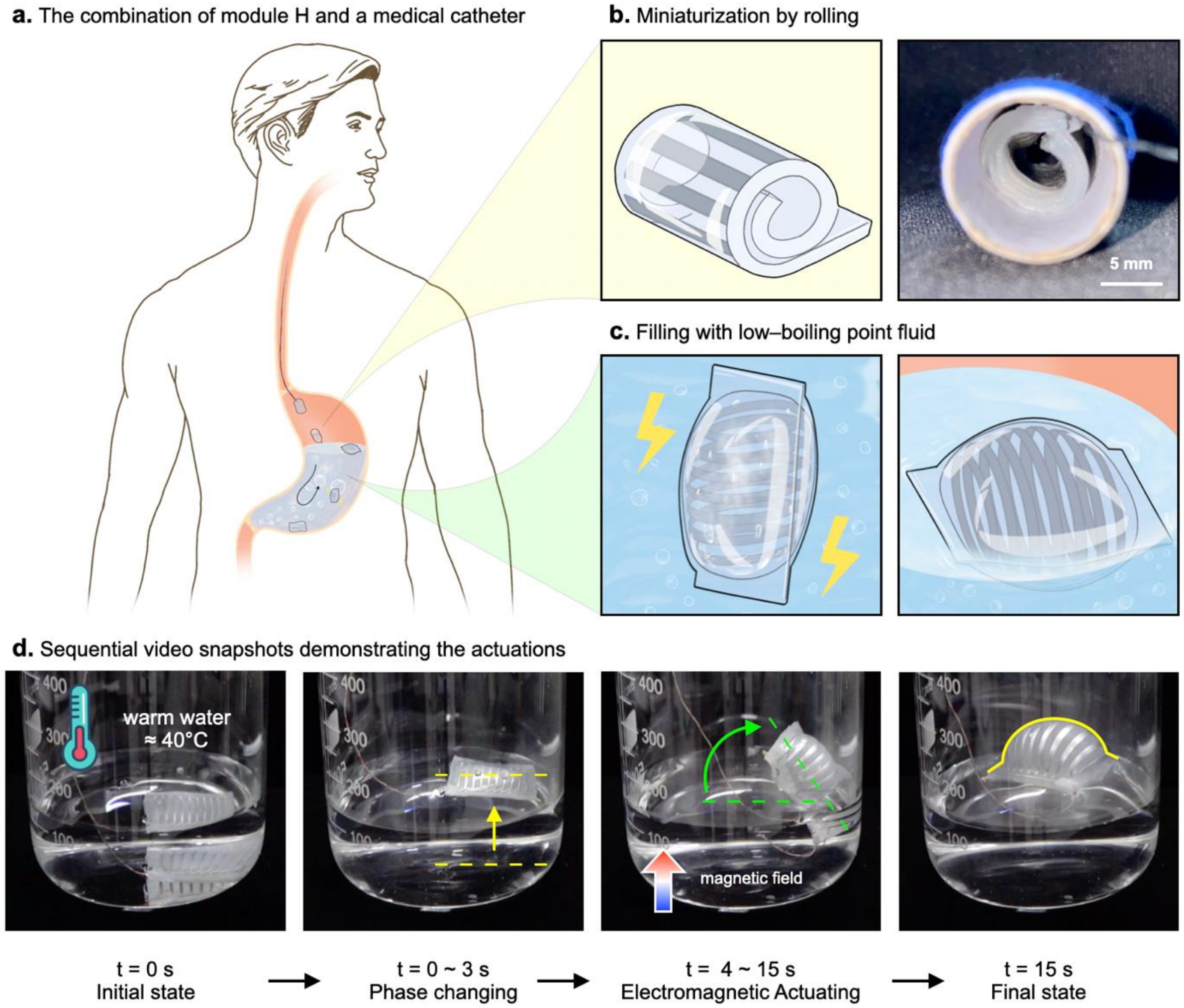

Supplementary Figure 34. The potential bioengineering application for module H by combining with a micro-catheter.

a Schematic of the combination of module H and a micro-catheter performing the gastric treatment. Illustrations include a folded module H that slides from the microcatheter, sinks, floats, and actuates. **b** Rolling up approach and photograph for further miniaturization. **c** Actuation strategy of pre-storing low boiling point fluid. The phase-changing temperature of the Novec 7000 (3M, United States) is 35 ℃, which can quickly reshape the profile of the soft electromagnetic actuator inside the human body without tubes. **d** Proof-of-concept demonstration. In warm water, module H can show fast shape morphing, states transforming, and powerfully padding.

2. Supplementary Tables

Supplementary Table 1. Parameters used in analytic calculations

Parameter	Symbol	Value	Unit
Half long axis of the elliptical profile	a	15.9	mm
Half of the maximum chord of the coil cross-section	o_iN	11.7	mm
Thickness of the module H with no inflation	t	2.8	mm
Half side length of the module H	w	16.5	mm
Cofficients of the function B_z for the plate magnet	m	-3.5711	-
	n	284.8	-
Average mass of the H samples	M	3.71	g
Gravitational acceleration	g	9.8	m s^{-2}

Supplementary Table 2. Comparison of experimental and FEM results on the profile dimension *h* of the inflated module H

V_a / *h*	0 ml	1ml	2 ml	3 ml	4 ml	5 ml	6 ml	7 ml
Experiment (mm)	2.8	6.76	10.11	13.29	15.41	17.18	18.63	19.88
FEM (mm)	2.8	6.57	9.73	12.88	15.09	16.33	17.0	17.71

Supplementary Table 3. Comparison of experimental and theoretical results on the critical flipping current during the steady rotation for the inflated module H

V_a / *I*	3 ml	4 ml	5 ml	6 ml	7 ml
Experiment (A)	1.9	1.6	1.4	1.3	1.1
Theory (A)	1.71	1.41	1.21	1.08	0.99

Supplementary Table 4. Comparison of the flexibility and operational dimensionality for different soft actuators and robots

Operation category	Actuation method (mode)*	Actuator No.	Signal No.	Reprogram-mability	Selective actuation	Control complexity **	Response time	Reference
**Continuous rolling**	Pneumatic	8	8	N	✓	Complex	s	(*1*)
	DEA	6	3	N	✓	Moderate (High voltage)	ms	(*2*)
	SMA	7	7	N	✓	Simple	s	(*3*)
	Light (LCE)	24	External field	N	✓	Moderate	1~10 s	(*4*)
	Heat (LCE) (unidirectional rolling)	5	External field	N	N	Moderate	1~10 s	(*5*)
	Magnetic field	1	External field	N	N	Complex	ms	(*6*)
	Magnetic field	1	External field	N	N	Complex	ms	(*7*)
	**Self-vectoring**	**1**	**1**	✓	✓	**Simple**	**ms**	**This work**
**Omnidirect-ional motion**	Pneumatic	3	3	N	✓	Complex	s	(*8*)
	Vacuum	3	3	N	✓	Complex	s	(*9*)
	Pneumatic+ Vacuum	4	4	✓	✓	Complex	s	(*10*)
	DEA	9	8	N	✓	Moderate (High voltage)	ms	(*11*)
	HASEL	3 groups	3	N	✓	Moderate (High voltage)	ms	(*12, 13*)
	Electric heat (LCE)	3	3	N	✓	Simple	min	(*14*)
	Light (hydrogel)	1	External field	N	✓	Moderate	1~10 s	(*15*)
	Magnetic field	1	External field	N	N	Complex	ms	(*16*)
	Magnetic field	1	External field	N	N	Complex	ms	(*17*)
	Electro-magnetic (only 4-directional rotations)	5	4	✓	✓	Simple	ms	(*18*)
	**Self-vectoring**	**2**	**2**	✓	✓	**Simple**	**ms**	**This work**

**Shape morphing**	Pneumatic<br>(1 mode: bulge)	1	1	N	N	Complex	s	(*19*)
	Pneumatic+<br>Vacuum<br>(4 modes: extension, bowl, arch, saddle)	13	5	✓ (moderate speed)	✓	Complex	s	(*10*)
	Electric heat (LCE)<br>(3 modes: dome, saddle, arch)	48	4	N	✓	Simple	min	(*20*)
	Heat (SMP)<br>(1 mode: bend)	6	External field	N	N	Moderate	1~10 s	(*21*)
	Swelling (hydrogel)<br>(1 mode: bend)	1	External field	N	N	Moderate	min	(*22*)
	Light and magnetic field<br>(1 mode: bend)	1	External field	✓ (slow)	✓	Complex	1~10 s	(*23*)
	Magnetic field<br>(1 mode: contraction)	1	External field	N	N	Complex	ms	(*7*)
	Magnetic field<br>(1 mode: bend)	1	External field	✓ (complex and slow)	N	Complex	ms~min	(*24*)
	Electro-magnetic<br>(1 mode: bend)	5	5	✓ (Fast)	✓	Simple	ms	(*18*)
	**Self-vectoring**<br>**(3 modes: bend, twist, bend and twist)**	**6**	**2**	✓ **(Fast)**	✓	**Simple**	**ms**	**This work**

N no, ✓ yes
* Mode means achievable shape morphing modes by a specific structure.
** The setup and accessory equipment for control system.

3. Supplementary Notes

3.1 Simulation of the Lorentz torque

First, we draw the three-dimensional spiral coils in SOLIDWORKS and sweep along the paths as simplified models to replace the multi-layer microchannel structures. For module V (as shown in Supplementary Figure 2), we adopt a bilayer composite structure to enhance the amplitude of the vertical electromagnetic vector. For each layer, the size of the cross-section rectangle is 0.8 mm * 0.4 mm; The two planar coils are connected at the center. For module H (as shown in Supplementary Figure 3 and Supplementary Figure 4), the pitch of the helix is 2.80 mm, and the number of turns is 10; The central axis of the elliptical section is always 23 mm, and the length of the minor axis changes with the are volume of the internal chamber. According to the experimental data, the short axis lengths of the coil cross-section corresponding to the infalting volumes of 3, 5, and 7 ml are 11.49, 15.38, and 18.08 mm, respectively. The width of the 3D path sweep, also the thickness of each layer of the liquid metal microchannel, is set to 0.4 mm.

Then, we establish and analyze the magnetic field in the finite element simulation software COMSOL Multiphysics. It consists of two approximately cubic magnets, 50*50*38 mm, and a spherical air domain with a radius of 200 mm. The origin of the coordinates is the center of the area between the two cubes. The constitutive magnetization model of the magnets is determined by the residual flux density $||\boldsymbol{B}_r||$ = 1.3 T.

$$\boldsymbol{B} = \mu_0\mu_{rec}\boldsymbol{H} + \boldsymbol{B}_r, \boldsymbol{B}_r = ||\boldsymbol{B}_r||\frac{\boldsymbol{e}}{||\boldsymbol{e}||} \tag{1}$$

where μ_{rec} means recovery permeability, determined by natural material (NdFeB). We define the direction vector of the residual flux as $\mathbf{e}$ = (0,0,1). The steady-state results and the spatial magnetic field are shown in Supplementary Figure 18. A unidirectional quasi-uniform magnetic field can be formed between two magnets with opposite magnetic poles, which is also used for our subsequent theoretical analysis.

Next, we can directly import the 3D models into the FEA (Finite Element Analysis) through the connection terminal between these softwares. Later, we also need to select the working plane and the fixed axis required for the force calculation to constrain different modules. We have customized the corresponding parameters of liquid metal EGaInSn in the "Material" section. The electrical model of the

coil is $J_c = \sigma E$, where the conductivity $\sigma = 3.46 * 10^{-6}$ S m^{-1}. The coil current I_{coil} changes from 0.5 A to 2 A with step 0.5 A. In the "Research" section, we choose the coil geometry analysis and the steady-state solver to calculate the torque. The physical field controls the mesh division, and manually divides the local details to optimize the mesh quality further. The selected size is "extremely refined." A steady state is chosen for magnetic field analysis. The solver is configured in a fully-coupled iterative mode FGMRES, in which the geometric multigrid will be assembled at all levels. In addition, we can add different smoothers to optimize the solution process. Finally, as shown in Supplementary Figure 20, in the "Results" section, the global calculation and the arrows represent the numerical value and spatial direction of the Lorentz torque.

3.2 Characterization of the Lorentz torque

In the experiment, as shown in Supplementary Figure 19, we used a 3D-printed fixture to fix the two magnets, and marked the corresponding scale on the surface. Different modules are placed in the central area of the magnetic field through suspension, and we can control the angle and direction of suspension to characterize various working conditions of each module. It includes module V, module H with three typical inflation volumes of 3, 5, and 7mL, their two orthogonal suspension modes, and corresponding tilt angles.

For the data measurement, we choose a high-precision force sensor (LSB200, 20g, FUTEK, United States) whose input force and output voltage maintain linear correlation. Before use, we use 5g, 10g, and 20g standard weights to calibrate its output. At the same time, the data acquisition system (DHDAS, Donghua, Shanghai, China) records the output voltage of the sensor with a 50-Hz sampling frequency. We finally obtain Lorentz torque by multiplying the measured force and radius of rotation.

3.3 Theoretical analysis of the module H on a plate magnet

When the inflated module H is located at the center surface of the plate magnet and excited by current *I*, its motion is determined by a combination of Lorentz forces (F_{Li} and F_{Ri}), Gravity (*Mg*), reaction force (F_N), and frictional force *f*, as shown in Supplementary Figure 23. F_{Li} and F_{Ri} are the equivalent resultant Lorentz forces exerted on the upper and bottom half parts of the *i*th coil (from left to right, *i*=1,2, …10), respectively. F_{Li} drive module H to rotate anticlockwise, and F_{Ri} are on the contrary. While the net Lorentz torque *T* generated by F_{Li} and F_{Ri} makes the module rotate clockwise.

The out profile (middle longitudinal section) of the inflated module H is an approximate ellipse which can be expressed as

$$\frac{x^2}{a^2}+\frac{z^2}{b^2}=1 \tag{2}$$

where 2*a is the long axis and equals (29+1.4*2) mm, the length of the chamber's longitudinal section equals 29 mm, the wall thickness of the chamber approximately equals 1.4 mm with ignoring the change of wall thickness after inflation. 2*b is the short axis which can be measured from the out profile dimension *h* of the module corresponding to different air volume (see Supplementary Figure15d).

As shown in Supplementary Figure 23, we establish a moving coordinate system *xGz* on the module. The x and z axes coincide with the long and short axes of the elliptical cross-section of the module, respectively. G is the center of gravity, its coordinates in the absolute coordinate system *XOZ* are (X_G, Z_G). *C* is the contact point between the module and the *X* axis. The *X*-axis can be seen as the tangent line of the elliptical cross-section of the module at contact point *C*. Then we can obtain:

$$x_d=\frac{\left(a^2-b^2\right)\sin\theta\cos\theta}{\sqrt{a^2\sin^2\theta+b^2\cos^2\theta}} \tag{3}$$

$$Z_G=\sqrt{a^2\sin^2\theta+b^2\cos^2\theta} \tag{4}$$

where x_d is the horizontal position of the contact point with respect to the center of gravity G, θ is the rotation angle of the module on the magnet surface.

The module's equation of motion can be derived following Newton's second law:

$$M\ddot{X}_G=f+\sum_{i=1}^{10}\left(F_{Ri}-F_{Li}\right) \tag{5}$$

$$M\ddot{Z}_G = F_N - Mg \quad (6)$$

$$\dot{L} = T\text{-}Mgx_d \quad (7)$$

where L is the angular momentum of the module about the contact point:

$$L = MR^2\dot{\theta} + J_C\dot{\theta} \quad (8)$$

Then taking the time-derivative of equation (6), giving

$$\dot{L} = (MR^2 + J_G)\ddot{\theta} + 2MR\dot{R}\dot{\theta} \quad (9)$$

where J_C and J_G are the moments of inertia of the mdoule about the contact point C and the center of gravity G, respectively. R is the radius of rotation about contact point C, which can be derived from geometrical relationship:

$$R = GC = \frac{ab}{\sqrt{a^2\cos^2\gamma + b^2\sin^2\gamma}} \quad (10)$$

where γ is the contact angle, the angle between the z axis and radius of rotation GC, which is related to the rotation angle θ by the following equation:

$$\gamma = \frac{\pi}{2} + \theta\text{-}\arctan\left(\frac{Z_G}{x_d}\right) \quad (11)$$

Combing Equations (3)–(11), the equation of dynamic rotation of module H can be obtained:

$$(MR^2 + J_G)\ddot{\theta} + 2MR\dot{R}\dot{\theta} + Mgx_d\text{-}T = 0 \quad (12)$$

Due to the inflated module H having a hollow chamber, and its outside shell consisting of different materials, incldouing sillicone and lquid metal, the actual value of J_G is hard to be obtained. Here we mainly focus on steady-state cases. When the module H rotates to a stable angle θ under the excitation of current I, equation (12) is reduced to:

$$T = Mgx_d \quad (13)$$

where T and x_d are both the functions of θ. To derive the correspondence between current I and stable rotation angle θ, the calculation of the net Lorentz torque T is the key. The detailed formulating process is given below.

First according to the characterization result of the plate magnet, as shown in Supplementary Figure 22, we find a cylindrical region ($Z \leq 40$ mm and $R \leq 30$ mm) on the top of the magnet. The magnetic field is approximately parallel and decreases linearly along the Z direction in this region, and the magnetic field magnitude in R direction is negligible. So the magnetic field in this region can be depicted by a linear fitting formula B_Z=mZ+n.

Under the action of this magnetic field, the Lorentz force analysis on coil i is shown in Supplementary Figure24. When a current I flows anti-clockwise in the coil, we take an infinitesimal segment Δs near any point Q on the coil. According to Ampere's rule, the components of Lorentz force exerted on this coil segment can be given as:

$$\Delta F_{Ri} = B_Z I \Delta y_i \tag{14}$$

$$\Delta F_{si} = B_Z I \Delta z_i \sin\theta \tag{15}$$

We know that ΔF_s are symmetric about the z'-axis, so they cancel each other out without affecting the rotation of the module in the XZ-plane. While ΔF_{Ri} and ΔF_{Li} are in the XZ-plane which act together to rotate the module.

We number the ten coils from left to right as 1,2, ⋯10, their positions on the x-aixs can be written as x_i=2.8×(i-5)-1.4, (i=1,2, ⋯10). The cross-section m-m′ of coil i are shown in Supplementary Figure 24b and c, we assume it composes of two symmetric circular arcs. The equation of the upper arc can be expressed as:

$$y_i^2 + (z_i + r_i - h_i)^2 = r_i^2 \tag{16}$$

where h_i and r_i are the maximum height and the radius of the ith coil, respectively.

$$h_i = o_i H = \sqrt{b^2 - \frac{b^2}{a^2} x_i^2}\text{-0.7} \tag{17}$$

$$r_i = \frac{h_i}{2} + \frac{o_i N^2}{2h_i} \tag{18}$$

where o_iN is the half of the maximum width of the coil.

Then the absolute height in the XZ coordinate plane of any point Q on the arc can be obtained by geometry relationship:

$$Z = y_G - x_i \sin\theta + z_i \cos\theta \tag{19}$$

So, the corresponding magnetic field intensity B_Z can be calculated by this height. The resultant Lorentz force on the upper half of the *i*th coil can be calculated by integration along the arc length:

$$F_{Ri} = 2\int_s B_z I \sin\beta ds = 2I\int_0^{o_i N} \left(mZ + n\right)\sin\beta\sqrt{1+(z_i')^2}dy_i \tag{20}$$

Then the total torque to the contact point *C* of the Lorentz force exerted on the upper half of the *i*th coil can be derived as:

$$T_{Ri} = 2I\int_s B_z Z \sin\beta ds = 2I\int_0^{o_i N} \left(mZ + n\right) Z \sin\beta\sqrt{1+(z_i')^2}dy_i \tag{21}$$

where β is the angle between magnetic field ***B*** and ***ds***, which can be obtain by the relation of spatial angular rotation:

$$\cos\beta = \cos\theta\sin\alpha \tag{22}$$

where $\tan\alpha = |z_i'|$ is the slope of the arc. Then according to the arc equation and the trigonometric function relation, we can obtain:

$$\sin\beta = \sqrt{\frac{(z_i')^2\sin^2\theta + 1}{(z_i')^2 + 1}} = \frac{\sqrt{r_i^2 - y_i^2\cos^2\theta}}{r_i} \tag{23}$$

The equation of the lower arc of the *i*th coil can be expressed as:

$$y_i^2 + (z_i - r_i + h_i)^2 = r_i^2 \tag{24}$$

Similarly, we can obtain the resultant Lorentz force F_{Li} and the total Lorentz torque T_{Li} to the contact point *C* exerted on the lower half of the *i*th coil. Finally, combing equations (2)-(4) and (16)-(24), the net Lorentz torque exerted on the module when subjected to current I can be calculated as:

$$T = \sum_{i=1}^{10}\left(T_{Ri} - T_{Li}\right) \tag{25}$$

Taking equations (3) and (25) into (13), we can obtain an explicit functional relation between current I and rotation angle θ, which can be solved using numerical integration.

$$\begin{gathered} Mg\frac{\left(a^2 - b^2\right)\sin\theta\cos\theta}{\sqrt{a^2\sin^2\theta + b^2\cos^2\theta}} = \\ \sum_{i=1}^{10}\left(I\int_0^{o_i N} 8\cos\theta\left[(Z_G - x_i\sin\theta)m + \frac{n}{2}\right]\left[(h_i - r_i)\sqrt{\frac{r_i^2 - y_i^2\cos^2\theta}{r_i^2 - y_i^2}} + \sqrt{r_i^2 - y_i^2\cos^2\theta}\right]dy_i\right) \end{gathered} \tag{26}$$

Actually, the profile of the module H is not a strict ellipse, there are two convex parts on both sides of the module, as shown in Supplementary Figure 25. When the module rotates slowly from the horizontal state, the contact point *C* moves slowly on the elliptical profile. Once the rotation angle reaches a critical value θ_c, the endpoint of the convex part begins to contact the surface. If the module continues to rotate, the center of rotation then switches from point *C* to endpoint *D*, which leads to increased gravitational torque with a longer moment arm. Thus, more significant Lorentz torque corresponding to a bigger current is needed to rotate the module. Through the analysis, we can deduce that the rotation angle increases with the current when the angle is below θ_c, after that the module will stay at that angle with the increase of current, until a big enough current rotates the module again.

The critical angle θ_c can be obtained by the geometric relation:

$$Z_G = \sqrt{a^2 \sin^2 \theta_c + b^2 \cos^2 \theta_c} = Z_{G1} = (\frac{t}{2}\cot\theta_c + w)\sin\theta_c \tag{27}$$

An angle equation with respect to θ_c can be derived:

$$(w^2\text{-a}^2)\tan^2\theta_c + tw\tan\theta_c + (\frac{t^2}{4} - b^2) = 0 \tag{28}$$

where 2**w* and *t* are the width and initial thickness of the module, respectively. Solving equation (28), we can obtain the critical angles of modules with different air volumes. The corresponding critical currents can be calculated by equation (26).

After the θ_c, the new moment arm of gravity is

$$x_{d1} = w\cos\theta - \frac{t}{2}\sin\theta \tag{29}$$

Then the relation between current I and rotation angle θ can be obtained by

$$T\text{=}Mgx_{d1} \tag{30}$$

Then the rotation angle increases with the current again until it reaches over 90 °under a critical current.

4. Supplementary Reference

1. Shah, D. S., Powers, J. P., Tilton, L. G., Kriegman, S., Bongard, J. & Kramer-Bottiglio, R. A soft robot that adapts to environments through shape change. *Nat. Mach. Intell.* **3**, 51-59 (2021).
2. Li, W. B., Zhang, W. M., Zou, H. X., Peng, Z. K. & Meng, G. A fast rolling soft robot driven by dielectric elastomer. *IEEE/ASME Trans. Mech.* **23**, 1630-1640 (2018).
3. Huang, X. et al. Chasing biomimetic locomotion speeds: Creating untethered soft robots with shape memory alloy actuators. *Sci. Robot.* **3**, eaau7557 (2018).
4. Wang, Z., Li, K., He, Q. & Cai, S. A light-powered ultralight tensegrity robot with high deformability and load capacity. *Adv. Mater.* **31**, 1806849 (2019).
5. Kotikian, A. et al. Untethered soft robotic matter with passive control of shape morphing and propulsion. *Sci. Robot.* **4**, eaax7044 (2019).
6. Hu, W., Lum, G. Z., Mastrangeli, M. & Sitti, M. Small-scale soft-bodied robot with multimodal locomotion. *Nature* **554**, 81 (2018).
7. Kim, Y., Yuk, H., Zhao, R., Chester, S. A. & Zhao, X. Printing ferromagnetic domains for untethered fast-transforming soft materials. *Nature* **558**, 274 (2018).
8. Fang, G. et al. Soft robotic manipulator for intraoperative MRI-guided transoral laser microsurgery. *Sci. Robot.* **6**, eabg5575 (2021).
9. Robertson, M. A. & Paik, J. New soft robots really suck: Vacuum-powered systems empower diverse capabilities. *Sci. Robot.* **2**, eaan6357 (2017).
10. Yang, B. *et al.* Reprogrammable soft actuation and shape-shifting via tensile jamming. *Sci. Adv.* **7**, eabh2073 (2021).
11. Chortos, A., Mao, J., Mueller, J., Hajiesmaili, E., Lewis, J. A. & Clarke, D. R. Printing reconfigurable bundles of dielectric elastomer fibers. *Adv. Funct. Mater.* **31**, 2010643 (2021).
12. Acome, E. et al. Hydraulically amplified self-healing electrostatic actuators with muscle-like performance. *Science* **359**, 61-65 (2018).
13. Mitchell, S. K. et al. An easy-to-implement toolkit to create versatile and high-performance HASEL actuators for untethered soft robots. *Adv. Sci.* **6**, 1900178 (2019).
14. He, Q., Wang, Z., Wang, Y., Minori, A., Tolley, M. T. & Cai, S. Electrically controlled liquid crystal elastomer–based soft tubular actuator with multimodal actuation. *Sci. Adv.* **5**, eaax5746 (2019).
15. Zhao, Y. et al. Soft phototactic swimmer based on self-sustained hydrogel oscillator. *Sci. Robot.* **4**, eaax7112 (2019).
16. Wu, S., Ze, Q., Dai, J., Udipi, N., Paulino, G. H. & Zhao, R. Stretchable origami robotic arm with omnidirectional bending and twisting. *Proc. Nat. Acad. Sci.* **118**, e2110023118 (2021).
17. Kim, Y., Parada, G. A., Liu, S. & Zhao, X. Ferromagnetic soft continuum robots. *Sci. Robot.* **4** eaax7329 (2019).
18. Mao, G. et al. Soft electromagnetic actuators. *Sci. Adv.* **6**, eabc0251(2020).

19. Siéfert, E., Reyssat, E., Bico, J. & Roman, B. Bio-inspired pneumatic shape-morphing elastomers. *Nat. Mater.* **18**, 24-28 (2019).
20. Liu, K., Hacker, F. & Daraio, C. Robotic surfaces with reversible, spatiotemporal control for shape morphing and object manipulation. *Sci. Robot.* **6**, eabf5116 (2021).
21. Van, Manen. T., Janbaz, S. & Zadpoor, A. A. Programming 2D/3D shape-shifting with hobbyist 3D printers. *Mater. Horiz.* **4**, 1064-1069 (2017).
22. Sydney, Gladman. A., Matsumoto, E. A., Nuzzo, R. G., Mahadevan, L. & Lewis, J. A. Biomimetic 4D printing. *Nat. Mater.* **15**, 413-418 (2016).
23. Liu, J. A. –C., Gillen, J. H., Mishra, S. R., Evans, B. A. & Tracy, J. B. Photothermally and magnetically controlled reconfiguration of polymer composites for soft robotics. *Sci. Adv.* **5**, eaaw2897 (2019).
24. Alapan, Y., Karacakol, A. C., Guzelhan, S. N., Isik, I. & Sitti, M. Reprogrammable shape morphing of magnetic soft machines. *Sci. Adv.* **6**, eabc6414 (2020).
