# Supplementary material for: Self-vectoring electromagnetic soft robots with high operational dimensionality
Source: Nat Commun. 2023 Jan 12;14:182. doi: 10.1038/s41467-023-35848-y (PMC9837125; doi:10.1038/s41467-023-35848-y)
Supplement: Supplementary file 3 — Description of Additional Supplementary Files [file 41467_2023_35848_MOESM3_ESM.pdf]

Description of Additional Supplementary Files

Supplementary Movie 1. High-dimensional and reprogrammable shape morphing of a trefoil-shaped SESR.

Supplementary Movie 2. Agile rolling locomotion on different rugged terrains of a single module H.

Supplementary Movie 3. Omnidirectional rotary movement and operation of a composite module H||H.

Supplementary Movie 4. Inflating, rotating, and swaying motions of the module H.

Supplementary Movie 5. Side to side flipping and continuous rolling of the module H.

Supplementary Movie 6. Reprogrammable shape morphing of a SESR with a symmetric H-H structure.

Supplementary Movie 7. Reprogrammable shape morphing of SESRs assembled by three H modules.

Supplementary Movie 8. Reprogrammable shape morphing of SESRs assembled by four H modules.

Supplementary Movie 9. Reprogrammable shape morphing of a SESR assembled by two composite V||H modules.

Supplementary Movie 10. Reprogrammable shape morphing of a SESR assembled by two H modules and one V module.

Supplementary Movie 11. Reciprocating crawling of a SESR assembled by two H modules in series.

Supplementary Movie 12. Turning and paddling locomotion of a SESR assembled by two H modules in parallel.

Supplementary Movie 13. Crawling and turning of a SESR assembled by a composite H||H module and another H module.

Supplementary Movie 14. Reciprocating crawling, folding, and flipping of a SESR assembled by an H module and a V module.

Supplementary Movie 15. Omnidirectional crawling and shape morphing of a SESR assembled by three H modules.

Supplementary Movie 16. Rotating and crawling of a SESR assembled by four H modules.

Supplementary Movie 17. Untethered padding SESR with control unit onboard.

Supplementary Movie 18. Underwater swimming SESR with tiny magnets onboard.

Supplementary Movie 19. Flating and actuation strategies by pre-storing low boiling point fluid.
